# MANNosylation of Mesoporous Silica Nanoparticles Modifies TLR4 Localization and NF‐κB Translocation in T24 Bladder Cancer Cells

**DOI:** 10.1002/adhm.202304150

**Published:** 2024-03-30

**Authors:** Mariam Hohagen, Laura Sánchez, Ann‐Jacqueline Herbst, Hanspeter Kählig, Jae Won Shin, David Berry, Giorgia Del Favero, Freddy Kleitz

**Affiliations:** ^1^ Department of Functional Materials and Catalysis Faculty of Chemistry University of Vienna Währinger Straße 42 Vienna 1090 Austria; ^2^ Division of Microbial Ecology Department of Microbiology and Ecosystem Science Centre for Microbiology and Environmental Systems Science University of Vienna Djerassiplatz 1 Vienna 1030 Austria; ^3^ Vienna Doctoral School in Chemistry (DoSChem) University of Vienna Währinger Str. 42 Vienna 1090 Austria; ^4^ Department of Organic Chemistry Faculty of Chemistry University of Vienna Währinger Straße 38 Vienna 1090 Austria; ^5^ Center for Nanomaterials and Chemical Reactions Institute for Basic Science (IBS) Daejeon 34141 Republic of Korea; ^6^ Core Facility Multimodal Imaging Faculty of Chemistry University of Vienna Währinger Straße 42 Vienna 1090 Austria; ^7^ Department of Food Chemistry and Toxicology Faculty of Chemistry University of Vienna Währinger Straße 38–40 Vienna 1090 Austria

**Keywords:** bladder cells, Caveolin 1, immunomodulation, mannose, MSNs, TLR4, UTIs

## Abstract

D‐mannose is widely used as non‐antibiotic treatment for bacterial urinary tract infections. This application is based on a well‐studied mechanism of binding to the type 1 bacterial pili and, therefore, blocking bacteria adhesion to the uroepithelial cells. To implement D‐mannose into carrier systems, the mechanism of action of the sugar in the bladder environment is also relevant and requires investigation. Herein, two different MANNosylation strategies using mesoporous silica nanoparticles (MSNs) are described. The impact of different chemical linkers on bacterial adhesion and bladder cell response is studied via confocal microscopy imaging of the MSN interactions with the respective organisms. Cytotoxicity is assessed and the expression of Toll‐like receptor 4 (TLR4) and caveolin‐1 (CAV‐1), in the presence or absence of simulated infection with bacterial lipopolysaccharide (LPS), is evaluated using the human urinary bladder cancer cell line T24. Further, localisation of the transcription factor NF‐κB due to the MANNosylated materials is examined over time. The results show that MANNosylation modifies bacterial adhesion to the nanomaterials and significantly affects TLR4, caveolin‐1, and NF‐κB in bladder cells. These elements are essential components of the inflammatory cascade/pathogens response during urinary tract infections. These findings demonstrate that MANNosylation is a versatile tool to design hybrid nanocarriers for targeted biomedical applications.

## Graphical abstract

Upon MANNosylation of dendritic mesoporous silica nanoparticles (DMSNs), stimulation of TLR4 can be observed in T24 bladder cancer cells. DMSN‐phenyl‐mannose materials returned patterns resembling receptor‐mediated internalization via endocytosis. Treatment with DMSN‐NCO‐mannose results in evenly distributed signals. Incubation of these materials with fimbriated and non‐fimbriated bacteria shows only selective adhesion of DMSN‐NCO‐mannose with fimbriated bacteria, and no selective adhesion for DMSN‐phenyl‐mannose.

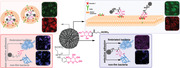

## 1 Introduction

D‐mannose is a common sugar (a monosaccharide isomer of glucose), which is described to hamper the adhesion of bacteria to the urothelium. D‐mannose naturally occurs in small amounts in various fruits such as cranberries, apples, and mangos as well as in foods such as egg white, soybeans, kidney beans, and peanuts; it is absorbed in the upper gastrointestinal tract and excreted in the urine.^[^ ^1^ ^]^ Mannose was found to be an effective supplement to treat acute urinary tract infections in women.^[^ ^2^ ^]^ Studies have shown that treatment of acute uncomplicated cystitis (AUC) via D‐mannose administration resulted in promising recovery rates, with an improvement of the symptoms within 3 days. Therefore, D‐mannose is often regarded as an effective alternative to antibiotics in the treatment of AUC, with a proposed mechanism of binding to the type 1 pili of enteric bacteria and thereby blocking their adhesion to uroepithelial cells.^[^ ^3^, ^4^, ^5^ ^]^

One emerging field to improve the treatment of UTIs is a form of immunomodulatory therapy in which non‐pathogenic probiotic bacteria are used to regulate, stimulate, or modulate the immune response.^[^ ^6^, ^7^ ^]^ An example of immunomodulatory effects of probiotics is the *Lactobacillus*‐mediated TLR therapy in which uropathogens are eliminated by activation of Toll‐like receptor 4 (TLR4). *Lactobacillus*‐mediated TLR therapy is based on the capacity of the TLRs to recognize pathogen‐associated molecular patterns (PAMPs) derived from bacteria lipopolysaccharide (LPS). LPS is a component of gram‐negative bacterial cells that initiates a cascade of protein–protein interactions, and, when recognized by TLR4, results in the production of pro‐inflammatory cytokines and interferons, and thereby, in an inflammatory and immune response.^[^ ^7^, ^8^, ^9^, ^10^ ^]^

Considering dietary habits that can affect inflammation and its resolution, high glucose intake and high fructose intake have also been proven to have pro‐inflammatory roles in inflammatory diseases.^[^ ^11^, ^12^, ^13^, ^14^, ^15^, ^16^ ^]^ Mastrocola et al. reported that increased protein levels of TLR4 were detected in the liver of fructose‐fed mice.^[^ ^11^ ^]^ Zhang et al. showed that high glucose‐fed mice had increased autoimmunity of colitis and experimental autoimmune encephalomyelitis (EAE) by promoting T helper‐17 (Th17) cell differentiation.^[^ ^16^ ^]^ Further, it was identified that D‐mannose was able to promote the activation of the latent form of TGF‐β, which required integrin αvβ8 and reactive oxygen species (ROS) in T cells, by inducting generation of T cells (Tregs) from native CD4+ T cells. These, in combination with the D‐mannose's ability to decrease glycolysis and thereby increase fatty acid oxidation in T cells, seemed to drive immune responses.^[^ ^16^ ^]^

In this work, we demonstrate that mannose‐modified materials can alter the localization of TLR4, making these materials plausible candidates for the regulation of the inflammatory response, which to the best of our knowledge, has not yet been exploited. At the molecular level, MANNosylation could be used as a tool to target mannose‐binding adhesins such as FimH, which is a protein at the tip of type I pili recognizing mannose, inhibiting the adhesion process of pathogenic bacteria in the early stages of infection.^[^ ^17^ ^]^ Barras and coworkers showed that nanodiamond (ND) particles modified with mannose are able to efficiently inhibit type 1 fimbriae‐mediated adhesion to eukaryotic cells with relative inhibitory potency.^[^ ^18^ ^]^ Along this line, Khanal et al. identified that the tri‐thiomannoside cluster‐conjugated NDs (ND‐Man_3_) showed not only potent inhibition of type 1 fimbriae‐mediated adhesion to yeast and T24 bladder cells but also to biofilm formation.^[^ ^19^ ^]^

Further, different MANNosylation strategies of MSNs were developed to utilize the biological activity of the sugar in combination with different chemical linkage to look at the already well‐studied mannose‐binding adhesins, which is essential for potential applications such as coatings of biomedical devices.^[^ ^20^, ^21^, ^22^, ^23^, ^24^ ^]^ To investigate the biocompatibility of the different formulations, we verified their cytotoxic potential in T24 bladder cancer cells and explored the response of crucial components of the inflammatory cascade, namely TLR4 and the transcription factor NF‐κB. In the respective pathway, TLR4 functions as a specific pattern recognition molecule and leads, together with myeloid differentiation factor 88 (MyD88), to activation of NF‐κB.^[^ ^25^, ^26^, ^27^, ^28^, ^29^ ^]^ Once activated, NF‐κB proteins can lead to the transcription of molecules (cytokines) which are essential for the immune response by recruiting immune cells to the infected site. Experiments were performed in the presence or absence of bacterial LPS stimulation. With this experimental layout, we observed that non‐functionalized MSNs, in the same mass concentration as MANNosylated particles, showed severe cytotoxicity compared to the mannose‐modified ones. In addition to reduced cytotoxic potential, the different chemical linkers used for the binding of mannose revealed a strong impact on TLR4 localization. To obtain further insights into the potential of MANNosylated materials to initiate an inflammatory cascade, the effect of MANNosylated materials in time intervals of 3, 6, and 24 h toward nuclear factor‐κB (NF‐κB) was investigated. NF‐κB belongs to the category of “rapid‐acting” primary transcription factors; and is therefore, essential in the regulation of cellular responses.^[^ ^25^, ^26^ ^]^ In this work, it was shown that MANNosylated materials were well‐tolerated by T24 cell line over 24 h. Further, these findings, together with the overall biocompatibility of the materials, support that TLR4 interaction seems functionally active; hence, it mirrored the activation of the NF‐kB transcription factor.

## 2 Results and Discussion

### 2.1 MANNosylation of Dendritic Mesoporous Silica Nanoparticles (DMSNs)

**Figure 1:**
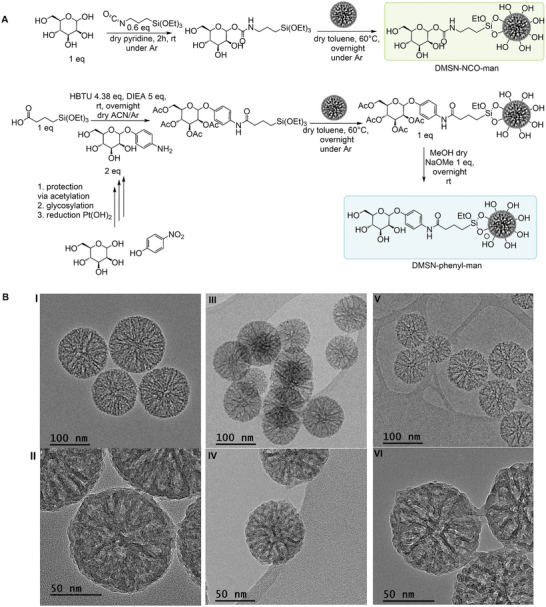
A) Reaction scheme for the two different MANNosylation methods. B) TEM images of the non‐functionalized native DMSNs (i,ii), DMSN‐NCO‐man (iii,iv), and DMSN‐phenyl‐man (v,vi).

Two different MANNosylation strategies were developed, following the procedure illustrated in **Figure** **1**A, using DMSNs depicted in Figure 1B‐i,ii. DMSNs were selected as nanocarrier because of their large pores, as well as their well‐controlled and uniform particle size distribution (average diameter of 100 nm), which were in agreement with previous works.^[^ ^30^, ^31^ ^]^ Surface modification of the MSNs with mannose was performed by first synthesizing the mannose‐silane and second, grafting the corresponding pre‐prepared mannose‐silane onto the surface of the native (as‐made) DMSNs via silane coupling/condensation. The mannose‐silane in the DMSN‐NCO‐man material was synthesized via a nucleophilic addition to the C═N bond in which the positively charged carbon of the isocyanate group (─N═C═O) was attacked by the nucleophilic hydroxy‐group, followed by proton shift to the negatively charged nitrogen atom, leading to the formation of a carbamate (Figure 1A). For DMSN‐phenyl‐man, first the mannose linker needed to be synthesized. The first step involved the protection of the hydroxy groups via acetylation, followed by glycosylation with p‐nitrophenol in the presence of BF_3_‐OEt_2_ as a catalyst, yielding the p‐nitrophenyl glycoside, which was reduced to an amino group catalyzed by Pd(OH)_2._ Finally, the phenyl‐mannose silane was synthesized via activation of the carbonyl moiety of the triethoxysilylbutanoic acid silane with a coupling reaction (HBTU) creating an active ester. The nucleophilic substitution reaction, due to the poor leaving group, led to the formation of an amide bond (Figure 1A). The percentage of organics grafted on the final materials (after template removal) was determined by thermogravimetric analysis (TGA) (**Table** **1**; Figure S1, Supporting Information).

**Table 1:** Physicochemical parameters of the non‐functionalized (extracted) DMSNs and MANNosylated DMSNs.

Material	DLS^a)^			N_2_ physisorption data^b)^			TGA^c)^
	*d* [nm]	PDI	Zeta potential [mV]	NLDFT mode pore size [nm]	Surface area / *S*_BET_ [m^2^ g^−1^]	Total pore volume / *V* [cm^3^ g^−1^]	Mass loss [%]
DMSN	100	0.2	−12.8	7–10	628	1.3	—
DMSN‐NCO‐man	111	0.3	−12	7.4–8.8	574	1.2	7
DMSN‐phenyl‐man	125	0.2	−18	7.3–9.3	328	0.7	9

^a)^ The particle size and zeta potential were measured using DLS;

^b)^ The specific surface area, *S*_BET_, pore volume (*V*), and pore size were obtained from N_2_ physisorption analysis (at −196 °C);

^c)^ Mass loss values were calculated in the temperature range of 150–700 °C by TGA.

To evaluate the porosity of the particles after MANNosylation, nitrogen physisorption measurements (at −196 °C) were performed (Figure S2, Supporting Information). The specific surface area (*S*_BET_), total pore volume, and mean pore size values are summarized in Table 1. A decrease in the *S*_BET_ and pore volume was observed with higher amount of grafted mannose. For DMSN‐NCO‐man having 7 wt% of organics, the specific surface area, as well as the pore volume, was reduced by about 8% (574 m^2^ g^−1^, 1.2 cm^3^ g^−1^) relative to the non‐functionalized material (628 m^2^ g^−1^, 1.3 cm^3^ g^−1^). In contrast, DMSN‐phenyl‐man with slightly higher grafting efficacy (9 wt%) showed a dramatic reduction of 46% of the porosity compared to the native (extracted) material, with significantly decreased *S*_BET_ (328 m^2^ g^−1^) and pore volume (0.7 cm^3^ g^−1^), which was most likely due to a pore blocking effect provoked by the bulkier nature of the phenyl‐man ligand. As observed by transmission electron microscopy (TEM) (Figure 1B‐i–vi), the DMSNs presented a dendrimer‐like mesopore organization, with apparently two populations of pores: smaller pores in the core and larger ones at the rim.^[^ ^31^ ^]^ The NLDFT pore size distributions calculated from the N_2_ physisorption isotherms corroborated these observations (Figure S2, Supporting Information). Due to the large pores of the DMSNs, pore condensation occurred at high relative pressure range, that is, 0.6–0.9, with a sharp capillary condensation step and hysteresis loop (Figure S2A, Supporting Information). The adsorption step at *P*/*P*_0_ above 0.9 was due to inter‐particle condensation, which is typically observed for particles in the size range of 100 nm.^[^ ^32^, ^33^ ^]^ The volume of N_2_ adsorbed at the lower pressure range was assigned to the presence of smaller pores of ≈7 nm in width (Figure S2B, Supporting Information), while a second maximum was centered at 10 nm for the non‐functionalized material. MANNosylation resulted mostly in a decrease of the second pore size compared to the pure silica material; however, pores remained large enough to accommodate possible guests. For DMSN‐NCO‐man, smaller pores with a width of ≈7.4 nm and bigger pores centered at 8.8 nm could be identified, whereas for DMSN‐phenyl‐man, pores with sizes of 7.3 and 9.3 nm were observed.

As the materials were prepared from the same parent material (non‐calcined DMSNs), the strong reduction of *S*_BET_ and total pore volume of DMSN‐phenyl‐man (9 wt% grafting) in comparison to DMSN‐NCO‐man (7 wt% grafting) was unexpected. To further understand this behavior, additional insights could be obtained from the TEM images of the MANNosylated materials (Figure 1B‐ii–vi). DMSN‐phenyl‐man, that is, the material in which the mannose was anchored to the phenyl‐silane linker via an amide bond, showed an interesting behavior of the attached mannose acting as connections/bridges between the particles (Figure 1B‐v,vi). This could contribute to the reduced porosity of the particle system. A similar behavior was reported by von Baeckmann et al. in the case of the grafting of inulin on other types of MSNs.^[^ ^34^ ^]^ On the other hand, in the case of the conjugation via carbamate formation (DMSN‐NCO‐man), the mannose attachment did not form pronounced particle interconnections (Figure 1B‐iii,iv).

**Figure 2:**
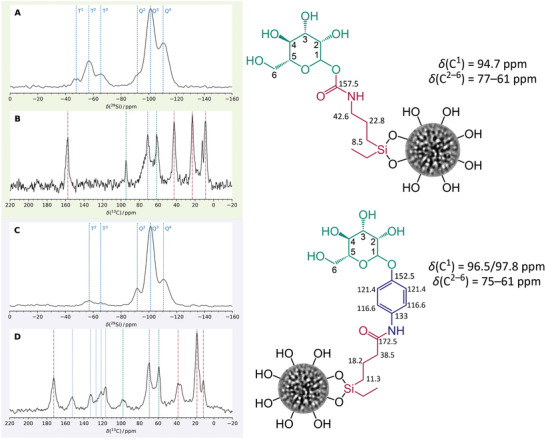
A,C) Solid‐state ^29^Si CP/MAS NMR spectra of MANNosylated materials DMSN‐NCO‐man and DMSN‐phenyl‐man, respectively. B,D) Solid‐state ^13^C CP/MAS NMR spectra of DMSN‐NCO‐man and DMSN‐phenyl‐man, respectively. The signals marked in green correspond to the units of the mannose molecule and those in red correspond to the silane linker, and in blue, for the spacer in the case of the DMSN‐phenyl‐man material. The particles are represented much smaller than their actual size for space reasons.

Solid‐state NMR spectroscopy was performed to validate the surface modification with mannose, as illustrated in **Figure** **2**A,C. T^2^ and T^3^ signals in the ^29^Si CP/MAS NMR spectra of DMSN‐NCO‐man and DMSN‐phenyl‐man indicated successful surface modification of the DMSNs with the silane‐mannose ligand. T^0^‐species, located at −47 ppm, showed the presence of non‐covalently attached silane‐NCO‐mannose (Figure 2A); however, no T^0^‐species were identified for DMSN‐phenyl‐man, suggesting that with this grafting method, covalent bonding of the mannose‐silane linker was more complete (Figure 2C). Comparison of the ^13^C CP/MAS spectrum of the mannose/DMSN's physical mixture with the MANNosylated materials revealed a deshielding effect for the anomeric signals up to 6 ppm due to glycosylation; the region of the sugar core signals stayed unchanged (Figure S3, Supporting Information). The successful addition (DMSN‐NCO‐man) and nucleophilic substitution (DMSN‐phenyl‐man) reactions could readily be monitored via ^13^C CP/MAS NMR due to the formation of methylurethane (157.9 ppm) or amide (172.5 ppm) moieties, respectively (Figure 2B,D).

With infrared spectroscopy, the characteristic absorbance band of *ν*(─NCOO─) of the formed carbamate moiety was observed in sample DMSN‐NCO‐man (Figure S4A, Supporting Information). For DMSN‐phenyl‐man, the formation of the amide was proven by its characteristic absorbance band of *ν*(─(C═O)─amide) (Figure S4B, Supporting Information). These findings additionally prove that the mannose grafting was successful. The colloidal stability was tested in PBS (pH 7) at 37 °C with dynamic light scattering (DLS) as a function of time (Figure S5A,B, Supporting Information). Initial particle size and zeta potential values are listed in Table 1. The hydrodynamic diameter of the DMSN‐phenyl‐man material was in average 13% bigger than that of the DMSN‐NCO‐man sample; however, for both materials, a consistently low polydispersity index (PDI) below 0.3 was observed. These findings indicate that the materials exhibit a narrow particle size distribution. Further, almost no change in the hydrodynamic diameter was observed after a period of 40–250 h. From this, one can conclude that MANNosylation strongly enhances the colloidal stability of the DMSNs, making them promising candidates for further biological profiling.

### 2.2 Biocompatibility

**Figure 3:**
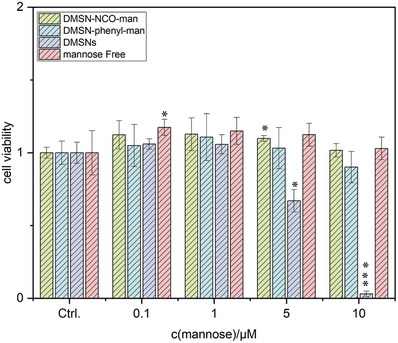
Cell viability assay in the presence of different MANNosylated particles as measured by metabolic activity (cell titer blue assay) in T24 cells after incubation for 24 h. Experiments were performed in technical quadruplicates in at least three independent cell preparations. * Indicates significant difference in comparison to controls (Ctrl.) (* *p* < 0.05; ** p < 0.01; *** *p* < 0.001). Data are expressed as T/C.

To start with the biological profiling of the MANNosylated materials, a cell viability assay (cell titer blue [CTB] assay) with T24 cell line was performed (**Figure** **3**). The cells treated with non‐modified and MANNosylated particles did not show any cytotoxicity in the concentration range of 0.1–1 µm(mannose), which translates to a particle concentration of 2.57–25.7 µg mL^−1^ for the non‐functionalized DMSNs. Interestingly, no reduction of cell viability was measurable when T24 cells were incubated with MANNosylated DMSNs to achieve a concentration of 5 µm mannose. However, when treated with non‐functionalized DMSNs, cell viability was reduced by 30% compared to the control. At the highest concentration of mannose‐functionalized material tested (10 µm mannose), cell viability was only slightly reduced by 10% (with the DMSN‐phenyl‐man material) whereas severe cytotoxicity (97% cell viability loss) was measured when cells were incubated with the non‐functionalized DMSNs (Figure 3). On the other hand, DMSN‐NCO‐man did not show any cytotoxicity within the tested concentration range. These findings suggest that particle modification has an impact on the biocompatibility, and even, reduces the toxicity of the silica particles in bladder cells. Considering that MSNs reduced cell viability only in their naked form, experiments were performed to independently validate this result and to exclude the possibility of unspecific effects, for example, related to the absorption of CTB dye. Immunofluorescence experiments (vide infra) confirmed cell damage and showed severe loss of morphology (Figure S6K–M, Supporting Information).

### 2.3 Cellular Uptake Followed by Live Cell Imaging

As the MANNosylated materials could be used as a drug carrier system, the potential of T24 cells to take up the particles intracellularly needed to be verified; for that, fluorescent‐labeled DMSNs (carrying 5 µm mannose) were generated. After incubation, 3D images of living cells were acquired. Indeed, even though UTIs are typically considered to be extracellular infections, it has been found that *Escherichia coli* strains are able to invade, replicate, and form intracellular bacterial communities within the murine bladder urothelium.^[^ ^35^ ^]^ With this experimental set‐up, it could be confirmed that both non‐functionalized DMSNs and MANNosylated particles could be taken‐up by the cells after 24 h of incubation (Figure S7, Supporting Information). Acquisition of 3D images combining z‐stacks across the whole cell height also enabled to confirm that in these experimental conditions, the particles did not aggregate, but rather, evenly distributed within the cytoplasmic compartment. To further underpin the specificity of the cell uptake mechanisms, experiments were performed using Pitstop 2. As an inhibitor for most of the endocytic pathways, Pitstop 2 interferes with the binding of proteins to the N‐terminal domain of clathrin (Figure S8, Supporting Information).^[^ ^36^ ^]^ In control conditions, it could be confirmed that both non‐functionalized DMSNs and MANNosylated particles were taken up when the N‐terminus of the clathrin domain was not blocked by Pitstop 2 (Figure S8A–D, Supporting Information). However, when the clathrin domain was blocked by Pitstop 2, fewer particles were taken up by the cells (Figure S8E–H, Supporting Information). These findings further corroborated that active cellular uptake occurred for MSNs, most likely via clathrin‐mediated endocytosis.

### 2.4 TLR4 and CAV‐1 Localization

Once cellular uptake and acceptable biocompatibility were confirmed, further experiments were performed to determine the effects of the MANNosylated materials on contributors of the inflammatory cascade: 1) the TLR4 receptor, which is a key effector of the inflammatory cascade owing to its ability to recognize the lipid A portion of the bacterial LPS molecule; while the carbohydrate core of the oligosaccharide promotes TLR4 translocation,^[^ ^37^ ^]^ and 2) caveolin‐1 (CAV‐1), which is essential in the regulation of caveolar endocytosis^[^ ^38^ ^]^ and whose activation has been described for TLR4 turnover in endothelial cells.^[^ ^39^ ^]^ Consistently, Jiao and coworkers also postulated that CAV‐1 phosphorylation regulates TLR4 signaling. They found that binding of Tyr14‐CAV‐1 to TLR4 is needed to activate TLR4 signaling production of pro‐inflammatory cytokines in endothelial cells.^[^ ^39^ ^]^

**Figure 4:**
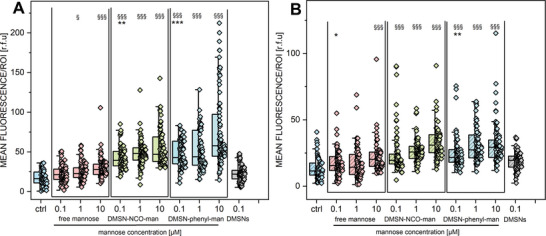
Quantification of the signal intensity of A) TLR4 and B) CAV‐1 performed after 24 h of incubation with free mannose, DMSN‐NCO‐man, and DMSN‐phenyl‐man in the concentrations of 0.1–10 µm [mannose] and non‐modified DMSNs (equivalent to 0.1 µm [mannose]) (2.57 µg mL^−1^). Data were obtained by the quantification of *n* > 25 regions of interest (ROIs) obtained from at least three independent cell preparations and expressed as mean fluorescence of relative fluorescence units (r.f.u.). * indicates significant difference in comparison to DMSNs in the concentration of 0.1 µm (* p < 0.05; ** p < 0.01; *** p < 0.001) and § indicates significant difference in comparison to the control (§ p < 0.05; §§ p < 0.01; §§§ p < 0.001). The complete statistical evaluation among the conditions can be found in Figure S9, Supporting Information. The significance was determined via one‐way ANOVA with Fisher LSD.

To study the role of TLR4 and CAV‐1 activation and the impact of surface chemistry of MANNosylated particles, immunofluorescence experiments were performed (Figure S6, Supporting Information). The T24 cells were treated with the two different mannose‐modified materials in comparison to free mannose for 24 h (0.1–10 µm) and including non‐functionalized DMSNs as the control. It was observed that the DMSN‐phenyl‐man material showed the highest increase in the signals associated with the immunodetection of TLR4 compared to the respective controls (**Figure** **4**A). MANNosylation triggered similar CAV‐1 signal increase throughout all concentrations (0.1–10 µm) compared to the controls (Figure 4B). These findings confirmed that the linking chemistry plays a substantial role in driving the biological response.^[^ ^40^ ^]^

### 2.5 TLR4 and CAV‐1 Profiling in LPS‐Stimulated T24 Cells

Once it was confirmed that MANNosylated materials could mobilize crucial effectors of the uptake/pro‐inflammatory cascade like CAV‐1 and TLR4, further experiments were performed to explore the response of T24 cells when incubated with MANNosylated particles and free mannose and mimicking the presence of an infection. Different LPS/material treatment strategies were carried out. Lipopolysaccharide (LPS) was used to simulate an infection because it was the most commonly found molecular component in the cell wall of gram‐negative bacteria and was able to stimulate the release of inflammatory cytokines, triggering inflammatory response toward pathogens by TLR4 activation.^[^ ^41^ ^]^ At first, a concentration‐response range finding study was performed to observe the kinetic and magnitude of the response of T24 cells to LPS (LPS concentrations of 1–100 ng mL^−1^ for 6–24 h). After 6 h incubation, the cells returned a concentration‐dependent response to LPS. The signals of TLR4 and CAV‐1 (Figures S10A,E and S11A,B, Supporting Information) increased significantly upon incubation with 1 ng mL^−1^ LPS and reached a maximum at the highest concentration tested. LPS incubation with 1 ng mL^−1^ for 24 h returned the highest TLR4 and CAV‐1 signal; however, T24 treated with LPS concentration of 10 or 100 ng mL^−1^ seemed to already show cytotoxicity (Figures S10B,F and Figure S11C,D, Supporting Information). Building on this, LPS concentration of 1 ng mL^−1^ was used for the further experimental set‐up.

**Figure 5:**
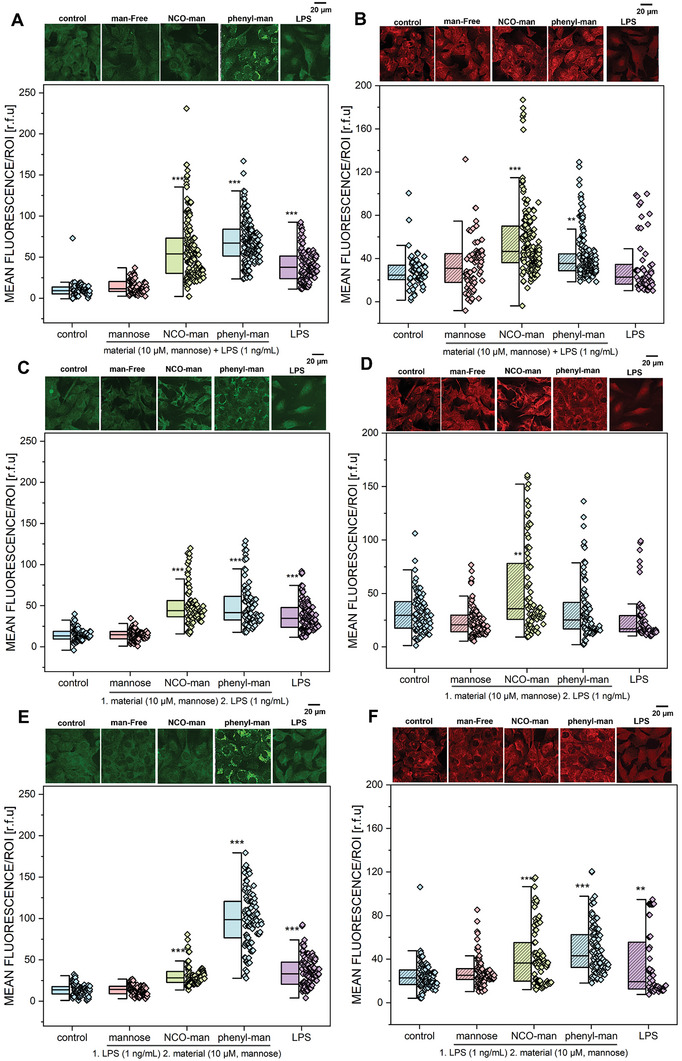
Representative images collected during the immunofluorescence experiments (TLR4, [A,C,E] in green and CAV‐1, [B,D,F] in red). Quantification of the signal intensity of TLR4 and CAV‐1 performed after 24 h of incubation with DMSN‐NCO‐man, DMSN‐phenyl‐man, free mannose, LPS, and controls. Characterization of the differential behaviors mimicking the presence of the infection via co‐incubation (material + LPS) (A,B), the presence of the material followed by the pro‐inflammatory treatment: 1. Material, 2. LPS (C,D), and finally, 1. LPS and 2. Material (E,F). Data were obtained by the quantification of *n* > 25 regions of interest (ROIs) obtained from at least three independent cell preparations and expressed as mean fluorescence of relative fluorescence units (r.f.u.). * indicates significant difference in comparison to the control (* p < 0.05; ** p < 0.01; *** p < 0.001). The complete statistical evaluation can be viewed in Figure S12, Supporting information. The significance was determined via one‐way ANOVA with Fisher LSD.

Three experimental models were tested within immunofluorescence experiments using TLR4 and CAV‐1 as references. The cell line T24 was treated with the two different mannose‐modified materials and free mannose, for 24 h (10 µm), including as controls non‐treated cells and LPS‐(1 ng mL^−1^) treated cells. As a first approach, co‐incubation (samples + LPS) (**Figure** **5**A,B) was performed, simulating a starting infection and treatment present at the same time. DMSN‐phenyl‐man + LPS triggered the highest signal associated with the immunodetection of TLR4 compared to the controls, followed by co‐incubation of DMSN‐NCO‐man + LPS (Figure 5A). Interestingly, DMSN‐NCO‐man showed a higher increase of CAV‐1 immunodetection compared to the LPS control, even though it did not show the highest TLR4 activation/signal (Figure 5B). The second model consisted first in the treatment with the material for 24 h and the subsequent incubation with LPS stimulation (Figure 5C,D). This model was chosen to explore the potential of MANNosylation as a possible formulation for biotechnological application as well as their prophylactic effect in the treatment of UTIs. Here, the application of DMSN‐phenyl‐man was less efficient in triggering the increase of TLR4 signal compared to the previous condition and DMSN‐NCO‐man showed the highest response. This supports the idea that different functionalization strategies could be more suitable than others for preventive applications or during acute infections. The last model involved incubation with LPS for 24 h, mimicking an infection and the following treatment with the mannose‐modified MSNs as it would be done in conventional UTIs treatment. In this scenario, DMSN‐phenyl‐man showed an essential increase in TLR4 signal as well as the highest values for CAV‐1 signal, compared to the controls (Figure 5E,F). However, the incubation with DMSN‐phenyl‐man did not only show an increase in signal associated with the immunodetection of TLR4 but also a different signal pattern, which seemed to be more intracellularly located and shaped like small vesicles, possibly related to the uptake or increased turnover of the receptor. Indeed, this would resemble the physiological behavior of TLR4, which upon binding of the LPS, is internalized via endocytosis;^[^ ^42^ ^]^ whereas, in comparison to the samples treated with DMSN‐NCO‐man and free mannose, the signal pattern was rather uniformly distributed. In summary, MANNosylated materials showed the highest stimulation of both TLR4 and CAV‐1 immunofluorescence signals compared to free mannose, supporting the potential of these mannose‐conjugated materials to interact with pathways related to inflammatory processes, such as those potentially triggered in case of UTIs. Further, it was identified that the linking chemistry used to decorate DMSNs with mannose did have a strong impact on the mode of action of those materials, which is essential for planning their biotechnological application.

At the molecular level, differences of the materials in biological response (i.e., TLR4 and CAV‐1 stimulation or/and selective mannose‐binding adhesion) could be a result of the mannose being linked via an aromatic spacer to the particles compared to simply by carbamate formation. Along these lines, it is possible to hypothesize differential interaction between the functionalized nanomaterials and TLR4 activation. LPS is a large glycolipid, which is composed of three structural domains: lipid A, the core oligosaccharide, and the O antigen. In case of an infection, it had been identified that the lipid A was known to interact with the binding pockets MD‐2 and MD‐2* of the TLR4 receptor.^[^ ^43^ ^]^ The interaction with those hydrophobic binding pockets was thought to be maintained by the alkyl chains of the A lipid. Further, it was identified that the inner core sugar interacted only with TLR4, and not with MD‐2‐MD‐2*, suggesting that the core sugars were not responsible for the endotoxic activity of LPS. This might be an indication that the additional interaction with TLR4 could be important for increasing LPS binding affinity and specificity to the formation of the MD‐2/TLR4‐ MD‐2*/TLR4* heterodimer.^[^ ^37^, ^44^, ^45^ ^]^ In addition, it was shown that the inner core sugars contributed to the TLR4‐mediated inflammatory responses.^[^ ^46^, ^47^, ^48^, ^49^, ^50^, ^51^ ^]^ Comparing the different domains of LPS, containing hydrophobic and hydrophilic moieties within its structure, to our MANNosylated particles, it could be argued that their general differences in TLR4 and CAV‐1 activation might be due to the different natures of the linkers. While the phenyl‐man‐material contains a phenyl spacer in addition to the hydrophobic propyl chain of the linker, the NCO‐man‐material contains in comparison less hydrophobic properties.

### 2.6 NF‐κB Nuclear Translocation

**Figure 6:**
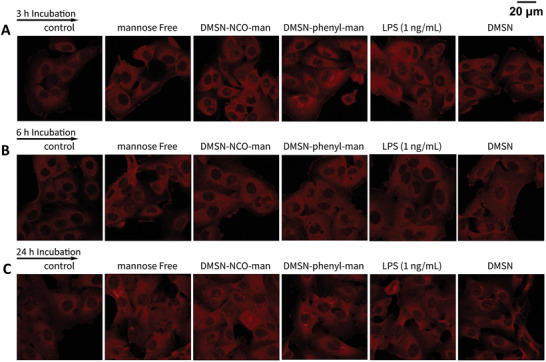
Representative images collected during the experiments of NF‐κB localization (in red); incubation with free mannose as the control, DMSN‐NCO‐man, DMSN‐phenyl‐man in a concentration of 10 µm [mannose], LPS (1 ng mL^−1^), and non‐modified DMSNs (equivalent to 0.1 µm [mannose]) (2.57 µg mL^−1^) for A) 3 h, B) 6 h, and C) 24 h.

**Figure 7:**
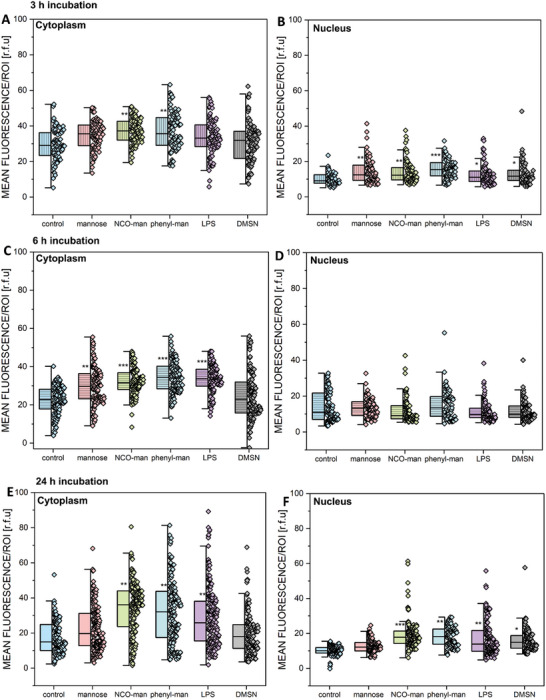
Quantification of the signal intensity of the NF‐κB transcription factor, in A,C,E) cytoplasm and in B,D,F) nucleus, was performed after 3 h (A,B), 6 h (C,D), and 24 h (E,F) of incubation with the control free mannose, DMSN‐NCO‐man, and DMSN‐phenyl‐man in a concentration of 10 µm [mannose], LPS (1 ng mL^−1^), and non‐modified DMSNs (equivalent to 0.1 µm [mannose]) (2.57 µg mL^−1^). The data were obtained by the quantification of *n* > 25 regions of interest (ROIs) obtained from at least three independent cell preparations and expressed as mean fluorescence of relative fluorescence units (r.f.u.). * indicates significant difference in comparison to the control (*p <0.05; **p <0.01; ***p <0.001). The complete statistical evaluation can be viewed in Figure S13, Supporting Information. The significance was determined via one‐way ANOVA with Fisher LSD.

We identified that MANNosylation favors interaction with the TLR4; therefore, further experiments were conducted to gain more insight into the potential of MANNosylated materials to activate inflammatory cascades^[^ ^52^ ^]^ The cell line T24 was treated with the two different mannose‐modified DMSNs and free mannose, for 3, 6, and 24 h (10 µm [mannose]), and non‐treated cells and LPS‐treated (1 ng mL^−1^) cells were included as negative and positive controls, respectively. Experiments were performed to evaluate the involvement of NF‐κB as transcription factor regulating inflammation. The NF‐κB localization pattern in the cytoplasmic and nuclear regions was similar for all time points tested (3, 6, and 24 h, **Figure** **6** and **Figure** 7).

MANNosylated materials modified the NF‐κB signal in the cytoplasm of T24 cells already after 3 h incubation. In this timeframe, all treatments significantly altered the localization of the transcription factor in the nuclear region (Figure **7**A,B). After 6 h incubation, regulatory events were limited to the cytoplasmic compartment (Figure 7C,D). Non‐functionalized DMSNs slightly increased the NF‐κB signal in the nuclear compartment after 24 h incubation (Figure 7F). After 24 h incubation, mannose‐treated cells were undistinguishable from controls, whereas MANNosylated materials and LPS maintained significantly high signal intensities for NF‐κB (Figure 7E,F), retracing for the materials the response profile already described for the immunolocalization of the TLR4 (Figure 4A; Figure S9A, Supporting Information). These findings, together with the overall good tolerability of the materials after 24 h treatment, support the view that TLR4 recruitment/relocalization could initiate functionally active cascades in T24 bladder cells, that is, reflecting on the activation of the NF‐kB translocation.

**Figure 8:**
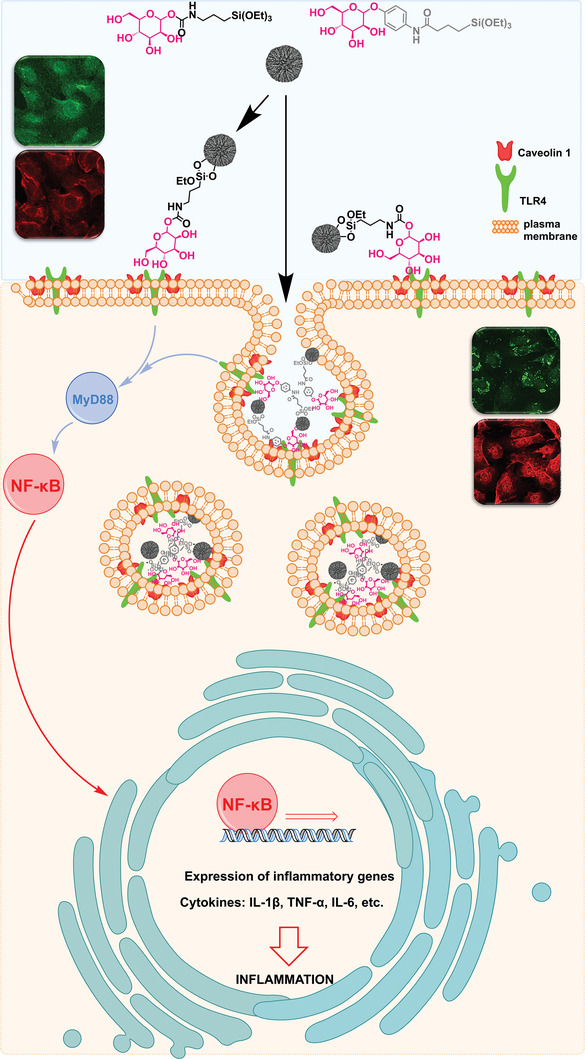
Schematic overview of particle functionalization and stimulation of the TLR4 receptor where the stimulation of DMSN‐phenyl‐man materials causes the formation of small vesicle‐like structures, possibly related to the uptake or increased turnover of the receptor. Whereas, when treated with DMSN‐NCO‐man, the signal pattern is rather evenly distributed. TLR4 translocation through the MANNosylated materials leads to the activation of NF‐κB proteins. The NF‐κB transcription factor in its inactive form is found in the cytosol and moves throughout activation into the nucleus, where the transcription of cytokines, responsible for activation of immune responses, is taking place.

To summarize, MANNosylation seems to affect TLR4, caveolin‐1, and NF‐κB in bladder cells. The relocalization of the TLR4 receptor caused by the mannose‐conjugated materials seems to trigger the activation of the NF‐κB transcription factor. The latter is the starting point for the transcription of cytokines and supports inflammation and immune responses, as schematically depicted in **Figure** **8**.

### 2.7 Attachment of DMSNs to Fimbriae‐Expressing Bacteria

**Figure 9:**
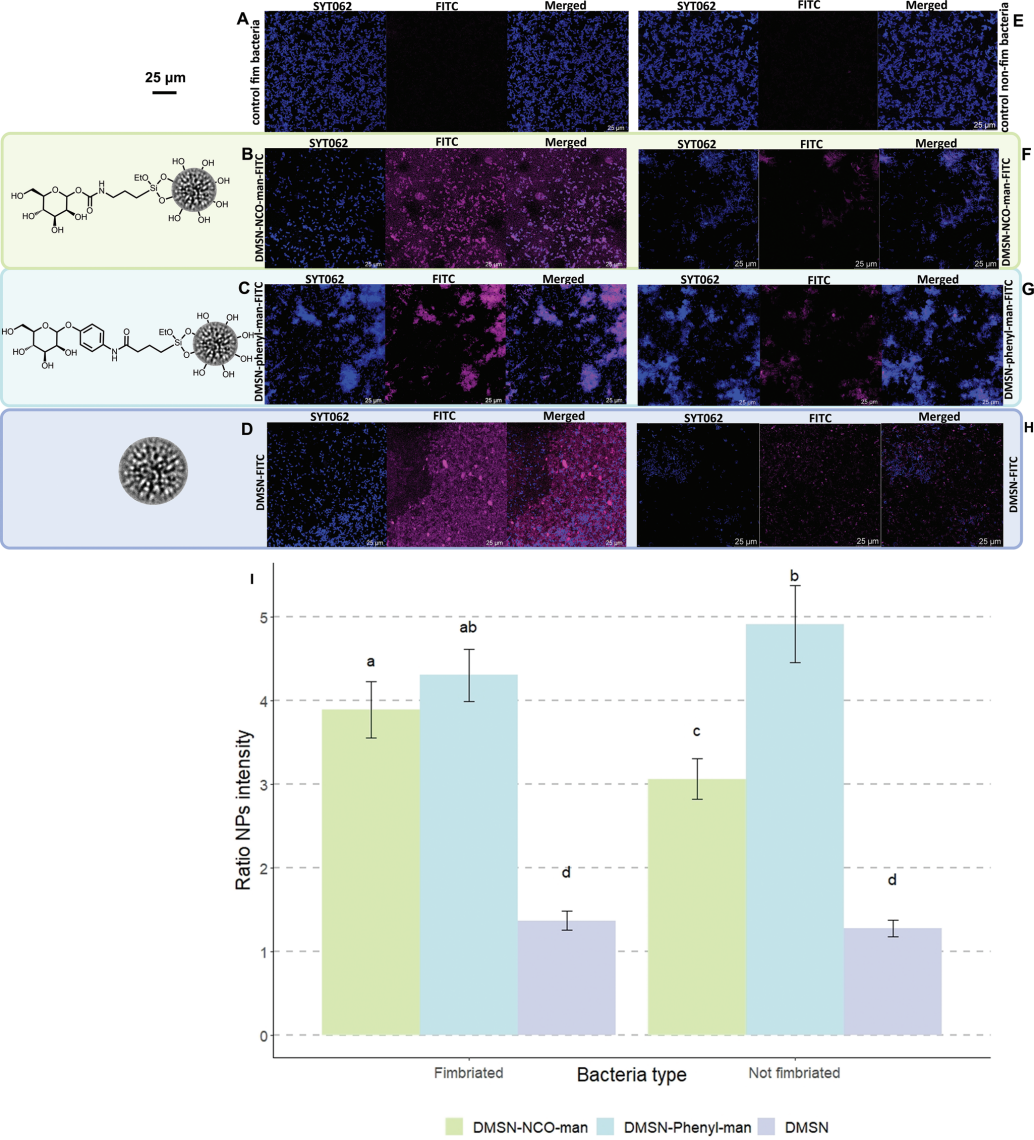
Representative fluorescence images of *S. enterica* expressing A) type 1 fimbrae alone, B) incubated with DMSN‐NCO‐man‐FITC, C) incubated with DMSN‐phenyl‐man‐FITC, and D) incubated with DMSN‐FITC, and E) *S. enterica* cells not expressing type 1 fimbrae alone, F) fimbriated bacteria incubated with DMSN‐NCO‐man‐FITC, G) DMSN‐phenyl‐man‐FITC, and H) DMSN‐FITC. Bacterial cells are represented in blue (SYTO 62) and DMSNs in magenta (FITC). Scale bars represent 25 µm. I) Attachment of DMSNs to fimbriated and non‐fimbriated bacteria measured by fluorescence spatial correlation. Bars represent the mean value of the ratio of fluorescence intensity between FITC signal co‐localized with SYTO 62 signal and FITC background signal. Error bars correspond to 95% confidence intervals. Letters (a–d) represent significantly different groups (*p* value < 0.05). Biological duplicates were used, and incubations were conducted in technical triplicates.

**Figure 10:**
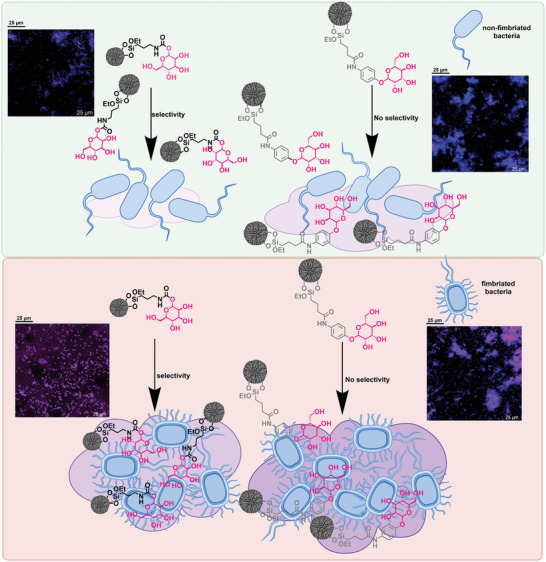
A) Schematic overview of the attachment of MANNosylated DMSNs to non‐fimbriated (light green [top]) and fimbriated (light red [bottom]) bacteria. The magnitude of adhesion is visualized by the size and the color intensity of a purple cloud. Incubation with DMSN‐NCO‐man shows a selective attachment to fimbriated bacteria whereas for the DMSN‐phenyl‐man material no selectivity is observed.

In parallel to the characterization of the effects of MANNosylated materials on bladder cells, experiments were performed also on prokaryotes. To verify the potential of mannose‐functionalized DMSNs to interact with bacteria, the specificity of the attachment of MANNosylated materials with type I fimbria‐expressing pathogenic model bacteria was explored with a co‐incubation assay. Fimbriated and non‐fimbriated *Salmonella enterica* cells were incubated with fluorescent‐labeled MANNosylated and non‐MANNosylated materials, and, after SYTO 62 staining to visualize the bacterial cells, confocal microscopy images were acquired (**Figure** **9**A–H). The microscopy images show that the bacteria tended to distribute uniformly in the control samples, when no particles were present, as well as in the presence of fluorescently tagged non‐functionalized particles (DMSN‐FITC). However, bacterial cells showed a quite different behavior in the presence of MANNosylated materials as they tended to cluster, suggesting an attraction caused by the mannose function (Figure 9A,B). Moreover, regions where only bacteria or particles are present were rare, which is a clear indication of the affinity of the bacteria for the mannose‐modified particles. Especially in the case of DMSN‐NCO‐man‐FITC, we observed that the particles were distributed on the surface of the slide in a way that spatially coincided with the signal of the bacteria in the SYTO62 channel, keeping them dispersed rather than inducing the formation of clusters (Figure 9B, single channels, and merged images). Thus, there was a distinct correlation between the localization of the DMSNs and bacteria (see **Figure** **10** for the experimental set‐up).

To substantiate these observations, the collected images were also used to perform calculations with the fluorescence intensity data (Figure 9I). The presence of nanoparticle binding to the bacterial surface was measured by ratio of the mean fluorescence value in the FITC channel of the particle‐covered to the uncovered surface, such that values ≈1 indicated a random distribution of the materials on the slide surface, and values greater than 1 indicated co‐localization of the materials and bacteria. A two‐way analysis of variance (ANOVA) was performed to compare the effects of MANNosylation and presence of Type 1 fimbria on the fluorescence intensity ratio, and homoscedasticity was subsequently confirmed. The test revealed that the presence of fimbria had a significant; although small, effect (F[1, 285] = 6.54, p = 0.011); while, the effect of the different MANNosylated materials and the interaction between them and fimbriation status was statistically significant and large (F[2,285] = 251.34, *p* <.001) and (F(2,285) = 12.09, *p* <.001, respectively). A Tukey post‐hoc test for multiple comparisons revealed significant pairwise differences between all groups, except for non‐MANNosylated materials with both fimbriated and non‐fimbriated bacteria, DMSN‐phenyl‐man with both types of bacteria, and both MANNosylated materials with fimbriated bacteria (*p* > 0.05).

The subsequent group‐wise comparison revealed that MANNosylated materials (a, ab, b, c) resulted in a clearly higher fluorescence ratio on average than naked materials (d) (Figure 9I), suggesting that MANNosylation can improve attachment of DMSNs to bacterial surface. Further, while DMSN‐phenyl‐man‐FITC did not show a significant difference between being incubated with fimbriated or non‐fimbriated cells (*p* = 0.057), probably due to an overall non‐specific attachment, this was not the case for DMSN‐NCO‐man‐FITC. The latter showed a significantly higher ratio of fluorescence intensity when incubated with fimbriated bacteria than when incubated with non‐fimbriated cells (*p* < 0.001), which was a sign of increased specificity in the attachment. Therefore, it could be concluded from these results that the MANNosylation of the materials significantly increased the affinity of bacteria for the nanoparticles with some specificity for fimbriated bacteria in the case of NCO‐linker functionalization. This suggests that there was potential for MANNosylated DMSNs to target specific bacteria expressing Type 1 fimbriae by adhering to and coating the cell surface. These findings further confirm the versatility provided by the different functionalization strategies in shaping the properties of the nanomaterials.

## 3 Conclusion

In this contribution, we elucidated two different MANNosylation methods for DMSNs and their consequences in terms of materials porosity and colloidal stability, as well as their influence on the biological activity profiling. The highest mannose grafting efficiency was achieved via amide formation using triethoxysilylbutanoic acid‐silane as coupling agent (phenyl‐man). Concerning porosity, the coupling via nucleophilic addition (NCO‐man) was most successful, showing only a reduction in specific surface area and pore volume of ≈8% compared to the starting material, whereas a strong reduction of porosity was observed in the DMSN‐phenyl‐man material. Both materials exhibited excellent colloidal stability and negligible cytotoxic effects even at high particle concentrations. The mannose modification even reduced the toxicity of the particles compared to native non‐functionalized DMSNs.

Moreover, this work emphasizes the importance of the linker chemistry, not only regarding the physico–chemical properties of the particles but also in the context of their in biological activity, which is essential for planning further applications, for example, treatment of UTIs. The two different MANNosylated materials showed distinct in vitro TLR4 and CAV‐1 signaling/recruitment behavior; and although further studies are required to explore the suitability of the materials for integrating medical treatments and therapeutic approaches, this clearly indicates interesting tunable biological effects. This could be useful for further development toward (nano)formulations with a loaded antibiotic, as well as for prophylactic treatment strategies.

Further, MANNosylation of the materials significantly increases the affinity of bacteria for the particles with some specificity for fimbriated bacteria in the case of NCO‐linker functionalization. This suggests a potential for MANNosylated DMSNs to target specific bacteria expressing Type 1 fimbriae by adhering to and coating the cell surface. These findings further confirm the versatility provided by the different functionalization strategies in shaping the properties of the nanomaterials. Thus, MANNosylation could be a promising strategy for possible biotechnological applications, including innovative drug formulations, coatings of biomedical devices, as well as non‐antibiotic alternatives.

## 4 Experimental Section

### Materials

Cetyltrimethylammonium chloride (CTAC, 25 wt% in H_2_O), tetraethylorthosilicate (TEOS, 99%), fluorescein isothiocyanate (FITC, > 90%), (3‐aminopropyl)triethoxysilane (APTES), (3‐isocyanatopropyl)triethoxysilane (95%), triethoxysilane, potassium tert‐butoxide, D‐mannose, acetic anhydride (99%), sodium bicarbonate (≥ 98%), ammonium carbonate (≥ 30.0%, NH_3_ basis), ethanol (puriss. p.a., absolute, ≥ 99.8%), hexane Reagent Plus (≥ 99%), and boron trifluoride etherate, and 4A molecular sieve were purchased from Sigma–Aldrich. L‐Lysine, (98%), sulfuric acid, 4‐nitrophenol (≥ 98%), and palladium hydroxide (99%), 2‐(1H‐Benzotriazol‐1‐yl)−1,1,3,3‐tetramethyluronium‐hexafluorophosphate (HBTU) (99%), dichloromethane (DCM), and acetonitrile (ACN) were purchased from Alfa Aesar. Diisopropylethylamine (DIEA) (99%), acrylic acid, hydrochloric acid (HCl) (37%), ethyl acetate (EtOAc) (≥ 99.5%), and platinum (IV) dioxide were purchased from Sigma–Aldrich. The cell culture and cytotoxicity experiment equipment were purchased from GIBCO Invitrogen (Karlsruhe, Germany), Lonza Group Ltd (Basel, Switzerland), Sigma–Aldrich Chemie GmbH (Munich, Germany), and Sarstedt AG&Co (Nuembrecht, Germany).

### Synthesis of Dendritic Mesoporous Silica Nanoparticles (DMSNs)

CTAC (8 mL) and L‐lysine (0.17 g) were dissolved in H_2_O (70 mL) under continuous stirring at 150 rpm (using a Heidolph stirring plate) at 60 °C. Subsequently, a mixture of *para*‐xylene (32 mL) and TEOS (8 mL) was added at a dropping rate of 76 mL h^−1^. The reaction mixture was stirred for 19 h at 60 °C. The organic phase was removed, and the material was separated from the fluid phase and collected after centrifugation at 10 000 rpm for 20 min. The resulting dendritic mesoporous silica nanoparticles (DMSNs) were then dried at room temperature (RT) overnight.

### Functionalization of the MSNs

The surface modification with mannose and particle labeling was performed under argon atmosphere using dry solvents and pre‐dried materials non‐calcined DMSNs. The nanoparticles were degassed under vacuum overnight at 70 °C. The nanoparticles were then suspended in dry toluene at 60 °C under inert atmosphere. After MANNosylation, the template was removed by solvent extraction through sonification in an EtOH/HCl mixture directly after the grafting procedure.

### Acetylation of Mannose

The acetylation of mannose was performed as reported by Sudibya et al.^[^ ^53^ ^]^ Briefly, D‐mannose (1 equiv., 11.1 mmol) was pre‐dried and dissolved in dry pyridine (7.5 equiv., 10 mL) at 0 °C. Subsequently, acetic anhydride (83.2 mmol) was added slowly drop‐wise and stirred overnight at RT. The reaction was quenched by the addition of ice water (100 mL) into the reaction flask. Then, the reaction solution was extracted three times with ethyl acetate (100 mL/50 mL/50 mL). The organic phase was washed with saturated sodium bicarbonate solution (2 × 70 mL) and (1 N) sulfuric acid (2 × 50 mL) and the volume reduced by evaporation (45 °C/250 mbar). The crude product (yellow oil) was purified by column chromatography: ethyl acetate/hexane (1/2), Rf = 0.22. The product: clear oil, yield: 96%.

### 4‐Nitrophenyl Glycoside Formation

The partially deprotected mannose was glycosylated as reported by Burke at al.^[^ ^54^ ^]^ In brief, the acetylated mannose (1 equiv., 9.52 mmol) was dissolved in dry DCM (20 mL) at RT under argon atmosphere. Pre‐dried 4A molecular sieves were added to the reaction mixture. 4‐nitrophenol (3 equiv., 28.5 mmol) was added and stirred at RT (1 h). Subsequently, trifluoride etherate (5 equiv., 47.5 mmol) was added and stirred at RT (48 h). The reaction was quenched with saturated sodium bicarbonate solution (20 mL, added slowly) and extracted with DCM (20 mL). The extracted organic phase was washed with 1 m sodium hydroxide solution and dried over sodium sulfate, filtered, and recrystallized in EtOAc/hexane at 4 °C. After recrystallization, an off‐white crystalline product was obtained (yield: 37%).

### Nitro‐Reduction of 4‐Nitrophenyl Glycoside

The nitro moiety was reduced as reported by Burke at al.^[^ ^54^ ^]^ In short, the 4‐nitrophenyl glycoside (1 equiv., 3.77 mmol) and palladium hydroxide (0.1 equiv., 0.377 mmol) were stirred in degassed DCM/methanol (6/4) (12 mL) in hydrogen atmosphere at RT (4 h). The palladium was removed via filtration (celite plug) and further purified via column chromatography: ethyl acetate/hexane (2/1), Rf = 0.52. A yellow oily product was obtained (yield: 48%)

### Synthesis of Triethoxysilylbutanoic Acid Silane

The synthesis method was performed as reported by Feinle et al.^[^ ^55^ ^]^ Acrylic acid (0.001 equiv., 0.05 mol) and platinum (IV) dioxide (1 equiv., 0.05 mmol) were stirred at 0 °C under argon atmosphere. Subsequently, triethoxysilane (0.001 equiv., 0.05 mol) was added slowly and the reaction mixture was stirred at 0 °C for 6 h. The reaction mixture was stirred overnight at RT. The crude mixture was filtered over a polytetrafluoroethylene syringe filter. The product obtained was a light brown colored liquid (yield: 76%).

### DMSN‐Phenyl‐Acetylated‐Mannose

To graft the protected mannose‐containing linker to the DMSNs, triethoxysilylbutanoic acid silane (1 equiv., 0.5 mmol) was activated with 2‐(1H‐Benzotriazol‐1‐yl)−1,1,3,3‐tetramethyluronium‐hexafluorophosphate (HBTU) (4.38 equiv., 2.19 mmol) and diisopropylethylamine (DIEA) (5 equiv., 2.5 mmol) by stirring in acetonitrile (ACN) (20 mL) for 20 min at RT. Subsequently the phenyl‐acetylated‐mannose (2 equiv., 0.99 mmol) was added and stirred at RT overnight. The DMSNs (0.23 g) were stirred for 3 h in toluene (20 mL) before the phenyl‐acetylated‐mannose linker reaction mixture was added to the particle suspension and stirred at 60 °C overnight. The light brown particles were centrifuged at 10 000 rpm for 20 min. The particles were washed with toluene and the surfactant extracted, as above. After extraction, the particles were dried at 40 °C overnight.

### DMSN‐Phenyl‐Mannose

The synthesis method was adapted from the procedure reported by Kong al.^[^ ^56^ ^]^ The DMSN‐phenyl‐acetylated‐mannose particles (1 equiv. 0.4 g) were suspended in dry MeOH (30 mL) for 2 h at RT. NaOMe (1 equiv., 0.052 mmol) was added to the suspension and stirred overnight at RT. The particles were centrifuged (20 min; 10 000 rpm) and the supernatant discarded. The material was washed twice with EtOH and was dried at 40 °C overnight. These particles were named DMSN‐phenyl‐man.

### DMSN‐NCO‐Mannose

Mannose (1 equiv., 8.32 mmol) was dissolved in pyridine (20 mL). 3‐isocyanatopropyl)triethoxysilane (1 equiv., 8.32 mmol) was added and stirred for 2 h at 50 °C. The pre‐dried DMSN material was stirred for 2 h in toluene at 60 °C before the NCO‐mannose reaction mixture was added. The reaction mixture was stirred overnight at 60 °C. These particles were named DMSN‐NCO‐man.

### Labeling of the DMSNs

FITC (4 equiv., 0.03 mmol) was dissolved in EtOH (5 mL). Then, APTES (1 equiv., 0.007 mmol) was added. The reaction mixture was stirred at RT under light protective conditions for 24 h. The pre‐dried material was stirred for 2 h (0.4 g in 120 mL toluene) before the APTES‐FITC ligand stock solution (80 µL) was added to the particle suspension. The suspension was stirred at 60 °C in the dark overnight. The material was recovered by centrifugation (20 min; 10 000 rpm), washed three times with EtOH, and dried at 35 °C in air for 24 h. The resulting labeled materials were named DMSN‐FITC, DMSN‐NCO‐man‐FITC, and DMSN‐phenyl‐man‐FITC.

### Colloidal Stability Tests

Colloidal stability of the MANNosylated materials was tested for several days at 36 °C. The particles (0.7 mg mL^−1^) were suspended in PBS buffer solution. The analysis of the dispersions was performed with a Malvern DTS Nano Zetasizer instrument via dynamic light scattering (DLS). The sample suspensions were kept under static conditions throughout the test, with no mixing or vortexing between the measurements.

### Materials Characterization

*Nitrogen Physisorption Analyses at −196 °C*: N_2_ physisorption isotherm measurements were performed with an Autosorb‐iQ3 sorption analyzer (Anton Paar, Boynton Beach, USA) at −196 °C (77 K). Before the analysis, the extracted and mannose‐modified DMSNs were outgassed at 150 °C and 60 °C, respectively, for 12 h. The Brunauer–Emmet–Teller (BET) method was used in the relative pressure range 0.05–0.2 *P*/*P*_0_ to calculate the specific surface area (*S*_BET_). The total pore volume was determined at *P*/*P*_0_ = 0.95. The pore size distributions (PSD) were calculated using the non‐local density functional theory (NLDFT, metastable adsorption branch) method considering a model of silica with cylindrical pores.

*Thermogravimetric Analysis (TGA)*: Coupled thermogravimetric analysis (TGA) and differential thermal analysis (DTA) measurements were performed with a Netzsch STA‐449 F3 Jupiter instrument under an airflow of 20 mL min^−1^ as carrier gas with a heating rate of 10°C min^−1^. The mass loss in percentage was given in the temperature range of 150–700 °C, to exclude the contribution of physisorbed water.

*Transmission Electron Microscopy (TEM)*: Transmission electron microscopy (TEM) images were obtained using a Titan G2 ETEM (FEI) at an accelerating voltage of 300 kV. The sample for TEM imaging was prepared by dropping a small amount of ethanol containing the suspended powder sample on a holey carbon film‐coated 300 mesh copper grid.

*Dynamic Light Scattering (DLS) and Zeta‐Potential Measurements*: Dynamic light scattering (DLS) studies were performed using a Malvern DTS Nano Zetasizer (equilibrium time 3 min, three measurements for each sample). 0.7 mg mL^−1^ of the material was dispersed in nanopure H_2_O. The suspensions were shaken and sonicated before the analysis. The zeta‐potential measurements were performed with the same instrument. Before zeta‐potential measurements, a standard solution with a zeta‐potential of −40 ± 5.8 mV was measured to ensure correct calibration.

*Solid‐State Nuclear Magnetic Resonance Spectroscopy (ssNMR)*: Solid‐state magic angle spinning nuclear magnetic resonance spectroscopy (MAS NMR) was conducted with a Bruker Avance NEO 500 wide bore system (Bruker BioSpin, Rheinstetten, Germany) using 4 mm triple resonance magic angle spinning (MAS) probe. Cross‐polarization (CP) was performed using a ramped contact pulse thereby sweeping the proton radio frequency field from 50% to 100%. For ^29^Si, the resonance frequency was 99.38 MHz, the MAS spinning speed was 8 kHz, and the CP contact time was 5 ms. The resonance frequency for ^13^C NMR was 125.78 MHz, the MAS rotor spinning was set to 14 kHz, and the CP contact time to 3 ms. ^1^H high‐power decoupling using SPINAL with 64 phase permutations was done throughout acquisition. The chemical shifts were reported in ppm and were referenced external for ^13^C to adamantane by setting the low field signal to 38.48 ppm, and for ^29^Si to sodium trimethylsilylpropane sulfonate (known as DSS) by setting the signal to 0 ppm.

*Fourier‐Transform Infrared Spectroscopy (FTIR)*: A Bruker Vertex 70 FTIR spectrometer equipped with Specac Golden Gate ATR accessory was used to obtain attenuated total reflectance Fourier‐transformed infrared (ATR‐FTIR) spectra. The spectra were recorded from the acquisition of 72 scans at 4 cm^−1^ resolution in the range from 4000 to 500 cm^−1^. Before each measurement, a background spectrum gathered from the acquisition of 72 scans at 4 cm^−1^ resolution was collected.

### Cell Cultures

The urinary bladder carcinoma cell line T24 (ATCC HTB4) was purchased from ATCC. The T24 cells were cultivated in McCoy's 5A medium (Gibco REF 22330‐021) supplemented with 10% v/v fetal calf serum, 1% v/v penicillin/streptomycin. The cells were incubated in a humidified incubator with 5% CO_2_ at 37 °C, as previously described.^[^ ^57^, ^58^ ^]^

### Cell Preparation and Standard Operating Procedure (SOP)

T24 were seeded in flat bottom 96 well plates at the concentrations of 20 000 cells per well. For the incubations, stock solutions of the mannose modified materials, DMSN‐NCO‐man (10 mm), DMSN‐phenyl‐man (10 mm), and free mannose (10 mm) were prepared in sterile H_2_O. The stock solutions were sonicated (15 min) and vortexed (20 s) before they were further diluted with sterile H_2_O to obtain concentrations of 5, 1, and 0.1 mm. The particles were resuspended before any cell experiment and further diluted (1:100) with McCoy's 5A medium. The cells were treated with 100 µL of each solution, followed by 100 µL of cell culture media supplemented with 20% v/v fetal calf serum 2% v/v penicillin/streptomycin to obtain the desired incubation concentrations and constant composition of the cell culture supplements. The treatments were conducted in technical quadruplicates obtained from at least three independent cell preparations (biological replicates). The cells were incubated for 24 h in the incubator (5% CO_2_ at 37 °C).

### Cell Titer Blue (CTB) Assay

The cell viability was assessed using the cell titer blue assay (CTB). For that, a fresh CellTiter‐Blue reagent solution was prepared before each experiment by diluting the CellTiter‐Blue reagent in DMEM (phenol red free) (1:10). After experimental incubation, the CTB solution was added to each well (120 µL) and incubated (5% CO_2_ at 37 °C) for 2 h. Subsequently, 50 µL of supernatant was removed from the wells and transferred into the corresponding wells of a black 96‐well plate. Finally, the fluorescence was measured at *λ* = 560Ex/590Em nm using a Cytation Imaging Multi‐Mode Reader (BioTek, Bad Friedrichshall, Germany).^[^ ^59^ ^]^ The signal ratio of the treated cells over the control was calculated in T/C. The data were represented as mean values ± the standard error and evaluated using at least three independent cell experiments in technical quadruplicates. Statistical analysis was performed with OriginPro 9.55 (OriginLab) applying one‐way ANOVA with Fisher's least significant difference (LSD) (threshold value *p* < 0.05).

### MSNs Uptake Experiments

T24 cells were seeded in 8 well IbiTreat slides at a density of 20 000 cells per well. The cells were treated with fluorescently labeled mannose modified materials with the concentration of 5 µm [mannose], whereas incubation with non‐functionalized DMSN was performed with the concentration equivalent to [1 µm mannose] (25.7 µg mL^−1^). The cells were incubated with silica nanoparticles for 24 h at 37 °C. The cells were washed (two times) with preheated (37 °C) live cell imaging solution and subsequently incubated with Cell Mask Deep Red plasma membrane stain for 15 min (dil. 1:2000). The cells were washed after incubation, live cell imaging solution added (200 µL), and the slide sealed. Imaging was performed using a Zeiss LSM710 laser scanning confocal microscope (ELYRA PS.1 system) equipped with a 63×/1.2 plan‐apochromat water immersion objective (Zeiss Microscopy GmbH, Germany).

### MSNs Uptake Pitstop 2 Experiment

The uptake Pitstop 2 experiment was conducted with minor modifications as reported by Iriarte‐Mesa et al.^[^ ^36^ ^]^ T24 cells were seeded in 8 well IbiTreat slides at a density of 20 000 cells per well. Subsequently, the clathrin inhibitor Pitstop 2 (8.25 mm in DMSO) was diluted in serum free medium (10 µm, 0.3% DMSO). The solution was added to the cells (200 µL per well), followed by 10 min‐incubation at 37 °C according to the specification of the supplier (Abcam Biochemicals, Cambridge, UK, Pitstop 2, ab120687). For the negative control (no Pitstop 2), 200 µL of serum free medium (including 0.3% DMSO) with no additional Pitstop 2 was used for the incubation. Subsequently, the cells were washed with serum free medium (two times), and the dispersions of FITC‐labelled modified and non‐modified nanoparticles were added to the corresponding wells. The cells were treated with MANNosylated particles with the concentration of 10 µm [mannose] whereas incubation with non‐functionalized DMSNs was performed in the concentration equivalent to 1 µm [mannose] (25.7 µg mL^−1^). The cells were incubated with silica nanoparticles for 6 h at 37 °C.

### Lipopolysaccharide (LPS) Treatment

Firstly, LPS concentration screening, identifying that LPS concentration of 1 ng mL^−1^ within an incubation time of 24 h returned the highest TLR4 and CAV‐1 signal (Figure S10, Supporting Information), was performed.^[^ ^60^ ^]^ Based on these findings, experiments were conducted following three different strategies. The cells were either treated with the materials and LPS at the same time, or the materials treatment was conducted first (24 h) followed by LPS stimulation (24 h). Lastly, the cells were first stimulated with LPS (24 h), followed by incubation with the MANNosylated materials (24 h). For all the layouts, the cells were fixed by addition of formaldehyde (3.7% in PBS, 15 min) and stained for immunofluorescence.

### Staining for Confocal Microscopy

For TLR4 and CAV‐1 immunofluorescence assay staining, samples were prepared as previously described with minor modification.^[^ ^61^ ^]^ The cells were treated with the materials with the concentrations (10, 5, 1, and 0.1 µm) for 24 h. After fixation with formaldehyde, the cells were permeabilized (0.2% Triton X‐100) and non‐specific binding sites were blocked with 2% donkey serum (2 h at RT). The cells were incubated with primary antibody mix including anti‐caveolin‐1 (1:300) and anti‐TLR4 (1:300) antibodies at +4 °C overnight. After several washing steps, the cells were incubated with secondary antibodies (Alexa Fluor 647 anti‐goat, Alexa Fluor 488 donkey anti‐mouse) for 1.5 h at RT. The antibodies were removed, the cells washed, mounted, and sealed with Roti‐Mount FluorCare with DAPI (Roth, Graz, Austria). For NF‐κB immunofluorescence assay staining, the samples were prepared with minor modification.^[^ ^62^, ^63^ ^]^ The cells were treated with the MANNosylated materials with the concentration (10 µm) for 3, 6, and 24 h. A non‐cytotoxic concentration of DMSN was added for comparison (2.57 µg mL^−1^; equivalent to 0.1 µm [mannose]). After fixation with formaldehyde, the cells were permeabilized (0.2% Triton X‐100), and non‐specific binding sites were blocked with 1% BSA in PBS‐A (60 min at RT). The cells were incubated with 647 Anti‐NF‐kB p65 antibody rabbit [E379] (Abcam) (1:400) at +4 °C overnight. After several washing steps, the cells were mounted and sealed with Roti‐Mount FluorCare with DAPI (Roth, Graz, Austria). Imaging was performed using a Zeiss LSM710 laser scanning confocal microscope (ELYRA PS.1 system) equipped with a 63X/1.4 plan‐apochromat oil immersion objective (Zeiss Microscopy GmbH, Germany). Image analysis was performed with the software ZEN 2012 Black Edition (Zeiss Microscopy GmbH, Germany). The relative fluorescence units (r.f.u.) were obtained applying constant acquisition parameters including signal/noise calibration and background correction. The assays were carried out in technical duplicates and three biological replicates. Statistical analysis was performed with OriginPro 9.55 (OriginLab) applying the one‐way ANOVA with the Fisher LSD test for the pairwise comparison. Distributions were considered statistically different using a threshold value of *p* < 0.05.

### Bacterial Cultures and Growth Conditions

*S. enterica* TH4038 [LT2 PfimZ51::Tn10dTc[del‐25] TcR; AnTc‐induced fimbriae production (fimZ)]^[^ ^64^ ^]^ and TH5698 [LT2 PfimZ51::Tn10dTc[del‐25] DfimA‐F6004::FRT TcR; fim operon deletion, control] bacterial strains were generously provided by Dr. Marc Erhardt from the Humboldt University of Berlin and stocked in 20% glycerol aliquots at −80 °C. Liquid cultures were routinely grown anaerobically in brain‐heart infusion supplemented (BHIs) medium (ThermoFisher, Austria) (5 g brain heart infusion, 12.5 g calf brain infusion, 2.5 g disodium hydrogen phosphate, 2 g glucose, 10 g peptone, and 65 g sodium chloride per liter [pH 7.2 to ≈7.4]. Adhesin expression was induced according to Hansmeier et al.:^[^ ^65^ ^]^ overnight cultures were diluted 1:30 into fresh medium containing 100 ng mL^−1^ anhydrotetracycline (AnTc) and incubated in anaerobiosis at 37 °C for 3.5 h in the dark.

### Nanoparticles Attachment Assay

After growth, the optical density of the cultures at 600 nm was measured and adjusted to 0.75 with 1× PBS. Cells were fixed with an equal volume of 70% ethanol in 1× PBS for 3 min; then, washed with PBS 1× and stained with 1 µL mL^−1^ SYTO 62 0.5 mm (Molecular probes, Germany) with a 10 min incubation at 37 °C in the dark. Nanoparticle solutions were prepared in PBS (10 mm). They were sonicated for 30 min; then, vortexed for 30 s immediately before use. The samples were prepared in a 96‐well plate, in a total volume of 200 µL per well. For each, either PBS (for the control samples) or cells were added in combination with 1.5 µL of the different DMSN solutions (namely DMSN‐FITC, DMSN‐NCO‐man‐FITC, and DMSN‐phenyl‐man‐FITC), when corresponding. The plate was incubated at 37 °C in the dark for 10 min, under agitation. Biological duplicates of the *S. enterica* strains were used, and incubations with the DMSNs were conducted in technical triplicates.

### Confocal Microscopy Visualization and Image Acquisition

For microscopy‐based evaluation of the bacteria‐nanoparticles interactions, 10 µL of each sample was spotted on a microscope slide and dried in the dark at 37 °C for 45 min. Samples were mounted with the non‐hardening and anti‐bleaching mounting medium CitiFluor AF1 (Science Services, Germany) and observed under a confocal scanning laser microscope (Leica TCS SP8X, Mannheim, Germany). Images of the samples and controls were acquired with a resolution of 1232 × 1232 to 1640 × 1640 pixels using a 93× glycerol objective (zoom factor = 1, pinhole size = 1). SYTO 62 was excited at a wavelength of 650 nm and detected in a range of 660–730 nm; FITC was excited at 495 nm and detected at 505–550 nm.

### Image Analysis and Statistical Analysis

Between 20 and 30 images per sample were collected and manually curated to analyze the homogenously dispersed samples using the software ImageJ. SYTO 62 channel was used to create a mask for the individual regions of interest (ROIs) corresponding to single cells. Fluorescence intensity of the integrated ROIs in the FITC channel was measured and divided by the mean fluorescence intensity of the background (region out of the ROIs mask). The mean of the fluorescence intensity of nanoparticles in contact with bacteria relative to dispersed nanoparticles was calculated using Rmisc package and plotted using ggplot2 package in RStudio (version 4.1.1). Error bars represent a 95% confidence interval for each sample. Statistical analysis was performed using one‐way ANOVA and Tukey's honestly significant difference (Tukey's HSD) post‐hoc analysis for pairwise comparisons (threshold *p* value < 0.05). Compact letter display (CLD)^[^ ^66^ ^]^ methodology was applied to identify statistically different factors among the variables.

## Conflict of Interest

The authors declare no conflict of interest.

## Supporting information

Supporting Information

## Data Availability

The data that support the findings of this study are available from the corresponding author upon reasonable request.
