# Supplementary material for: MANNosylation of Mesoporous Silica Nanoparticles Modifies TLR4 Localization and NF‐κB Translocation in T24 Bladder Cancer Cells
Source: Adv Healthc Mater. 2024 Mar 30;13(17):2304150. doi: 10.1002/adhm.202304150 (PMC11468387; doi:10.1002/adhm.202304150)
Supplement: Supplementary file 1 — Supporting Information [file ADHM-13-2304150-s001.pdf]

ADVANCED HEALTHCARE MATERIALS

Supporting Information

for *Adv. Healthcare Mater.*, DOI 10.1002/adhm.202304150

MANNosylation of Mesoporous Silica Nanoparticles Modifies TLR4 Localization and NF-κB Translocation in T24 Bladder Cancer Cells

Mariam Hohagen, Laura Sánchez, Ann-Jacqueline Herbst, Hanspeter Kählig, Jae Won Shin, David Berry, Giorgia Del Favero and Freddy Kleitz**

Supporting Information

MANNosylation of Mesoporous Silica Nanoparticles Modifies TLR4 Localization and NF-κB Translocation in T24 bladder Cancer Cells

Mariam Hohagen,[a] Laura Sánchez,[b] Ann-Jacqueline Herbst,[a, c] Hanspeter Kählig,[d] Jae Won Shin,[e] David Berry,[b] Giorgia Del Favero,[f, g] Freddy Kleitz*[a]*

[a] Department of Inorganic Chemistry – Functional Materials, Faculty of Chemistry, University of Vienna, Währinger Straße 42, 1090 Vienna, Austria

[b] Division of Microbial Ecology, Department of Microbiology and Ecosystem Science, Centre for Microbiology and Environmental Systems Science, University of Vienna, Djerassiplatz 1, 1030 Vienna, Austria

[c] Vienna Doctoral School in Chemistry (DoSChem), University of Vienna, Währinger Str. 42, 1090 Vienna, Austria.

[d] Department of Organic Chemistry, Faculty of Chemistry, University of Vienna, Währinger Straße 38, 1090 Vienna, Austria

[e] Center for Nanomaterials and Chemical Reactions, Institute for Basic Science (IBS), Daejeon 34141, Republic of Korea

[f] Core Facility Multimodal Imaging, Faculty of Chemistry, University of Vienna, Währinger Straße 42, 1090 Vienna, Austria

[g] Department of Food Chemistry and Toxicology Faculty of Chemistry, University of Vienna, Währinger Straße 38–40, 1090 Vienna, Austria

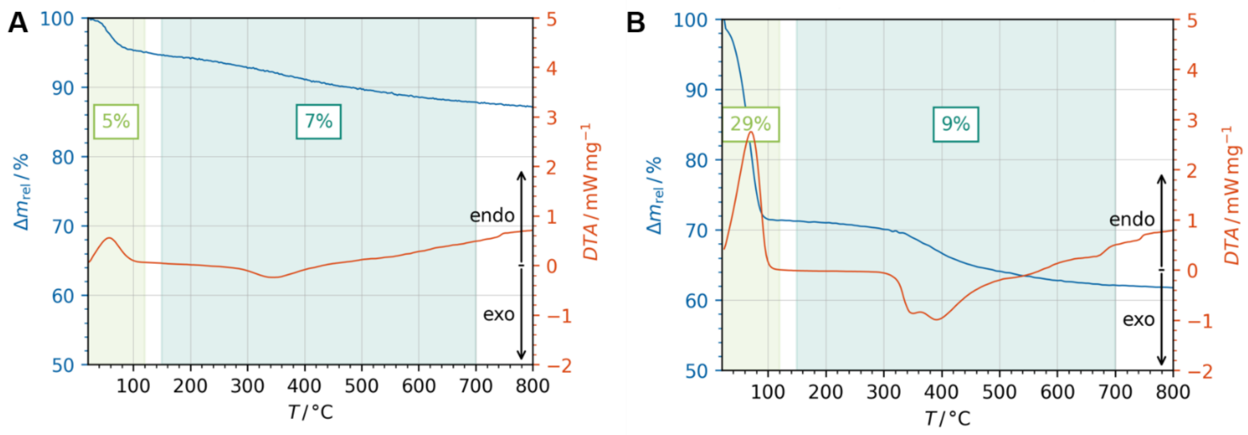

Figure S1. Thermogravimetric and differential thermal analysis (TGA-DTA) of DMSNs conjugated with mannose: DMSN-NCO-man (A) and DMSN-phenyl-man (B). The mass loss (blue line) was determined from 150 to 700 ºC (dark green area) to exclude contributions from remaining solvents or water (light green area).

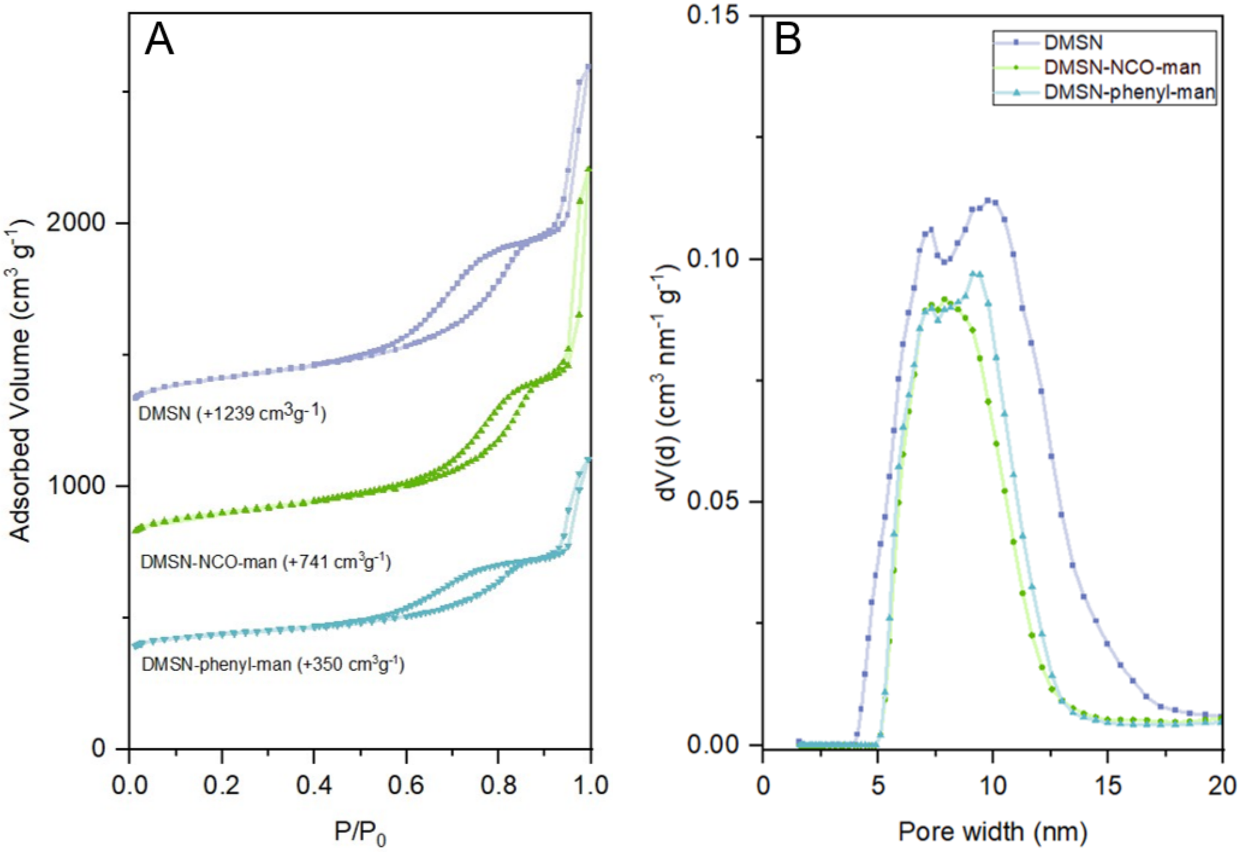

Figure S2. N_2 physisorption isotherms (measured at - 196 °C) of the different DMSNs, as indicated, (A) and their respective NLDFT pore size distributions (B).

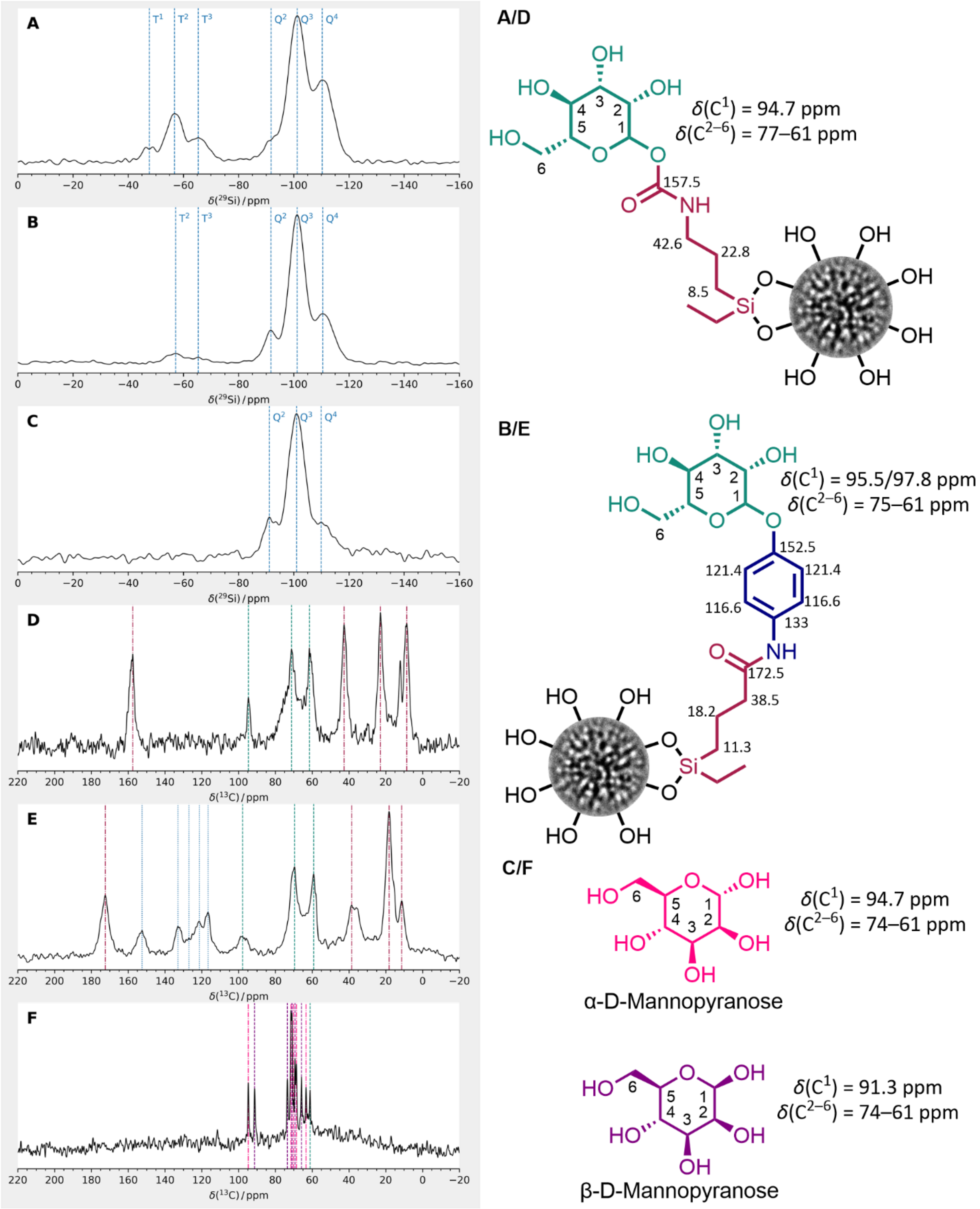

Figure S3. Solid-state ^{29}Si and ^{13}C CP/MAS spectra of the MANNosylated materials: ^{29}Si: DMSN-NCO-man (A), DMSN-phenyl-man (B), and physical mixture of mannose and native DMSNs (C); ^{13}C: DMSN-NCO-man (D), DMSN-phenyl-man (E), and physical mixture of mannose and native DMSNs (F). For the free mannose in the physical mixture, the chemical shifts originating from its α epimer are marked in pink and those of the β epimer are marked in purple. Common signals are visualized in green.

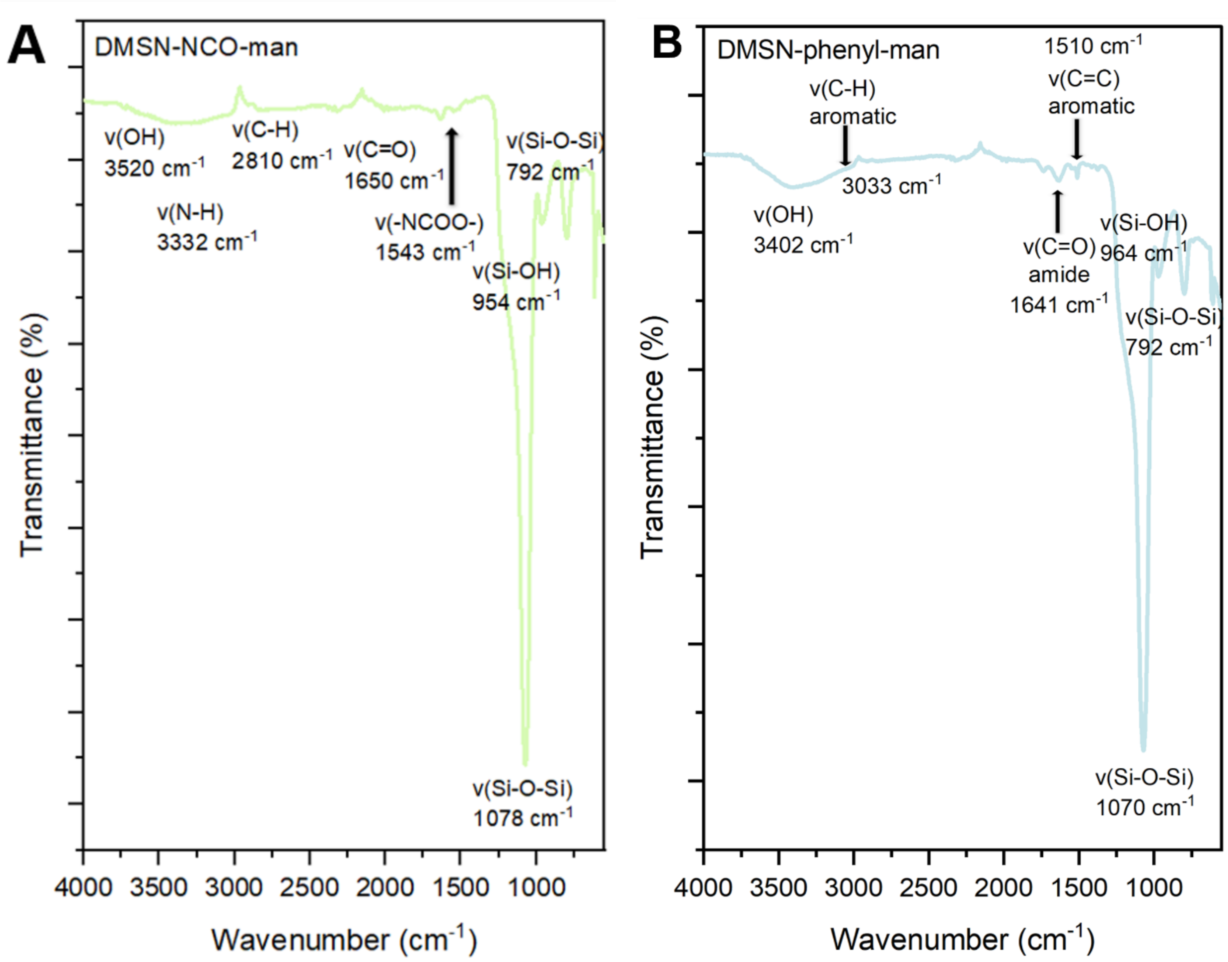

Figure S4. Attenuated total reflectance infrared (ATR-IR) spectra of mannose-functionalized DMSNs: DMSN-NCO-man (A) and DMSN-phenyl-man (B).

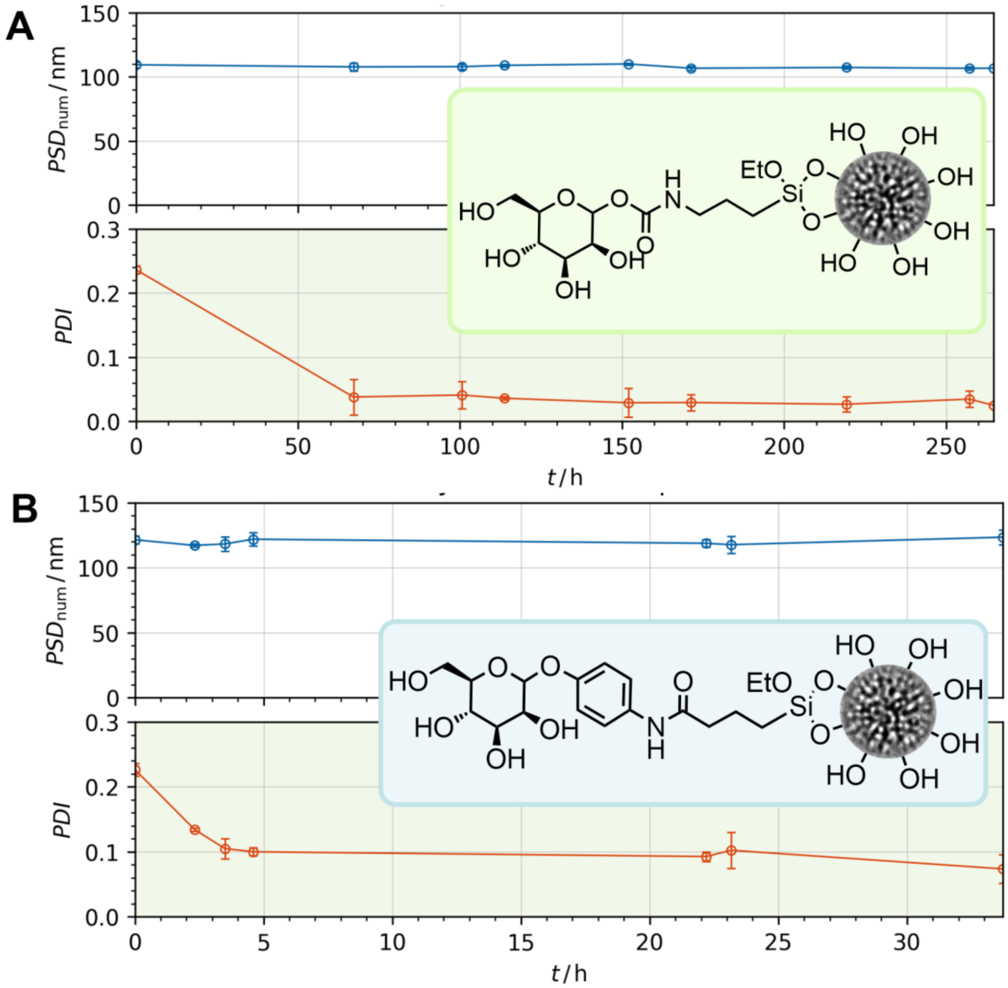

Figure S5. Colloidal stability tests *via* dynamic light scattering (DLS) analyses: DMSN-NCO-man (A) and DMSN-phenyl-man (B).

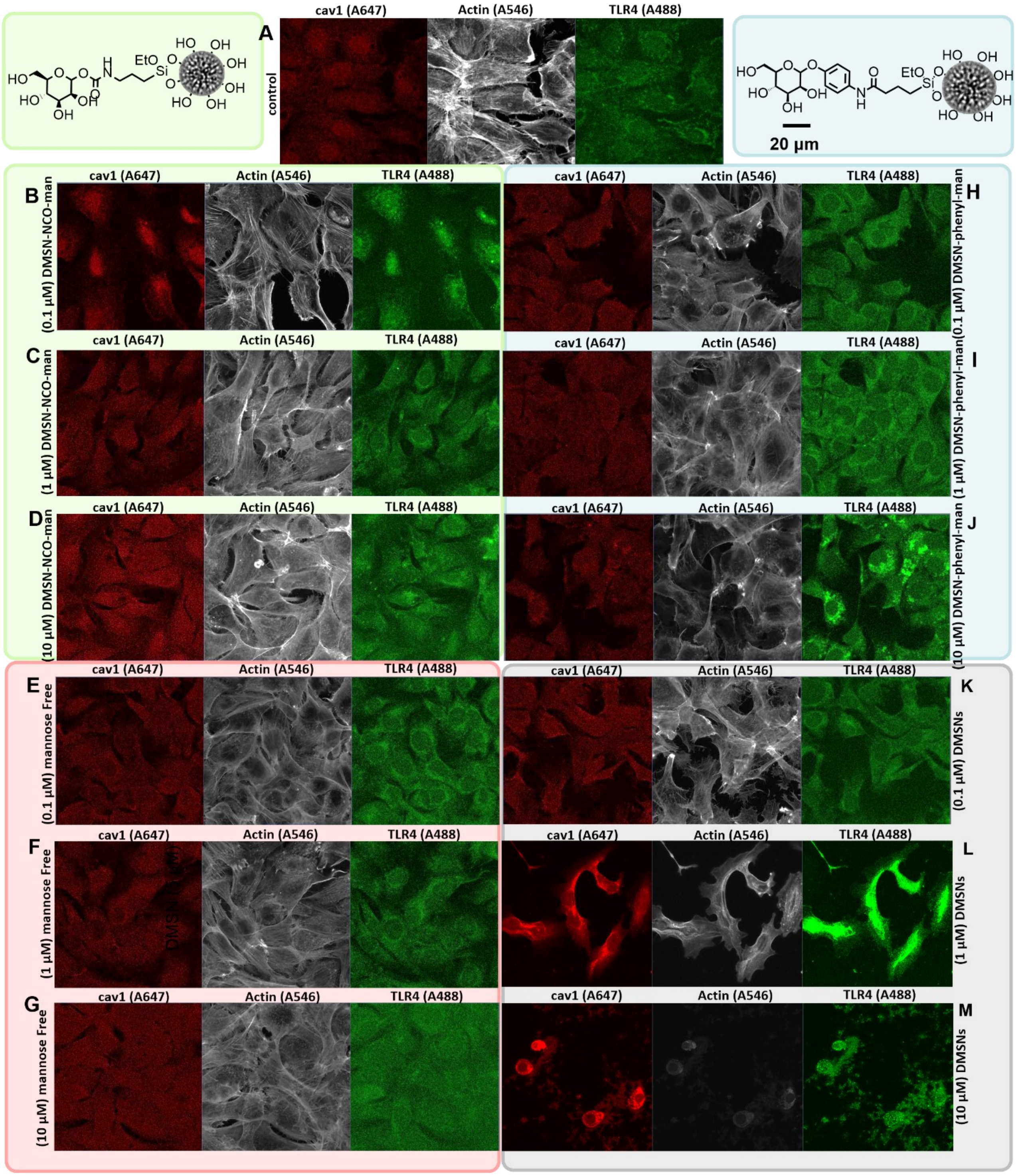

Figure S6. Representative images of immunofluorescence experiments performed after 24 h incubation with the treatments of interest and followed by immunodetection of CAV-1 (depicted in red), Actin (depicted in white), and TLR4 (depicted in green). Controls (A), DMSN-NCO-man (B-D), free mannose (E-G), DMSN-phenyl-man (H-J), as well as non-functionalized DMSNs (K-M).

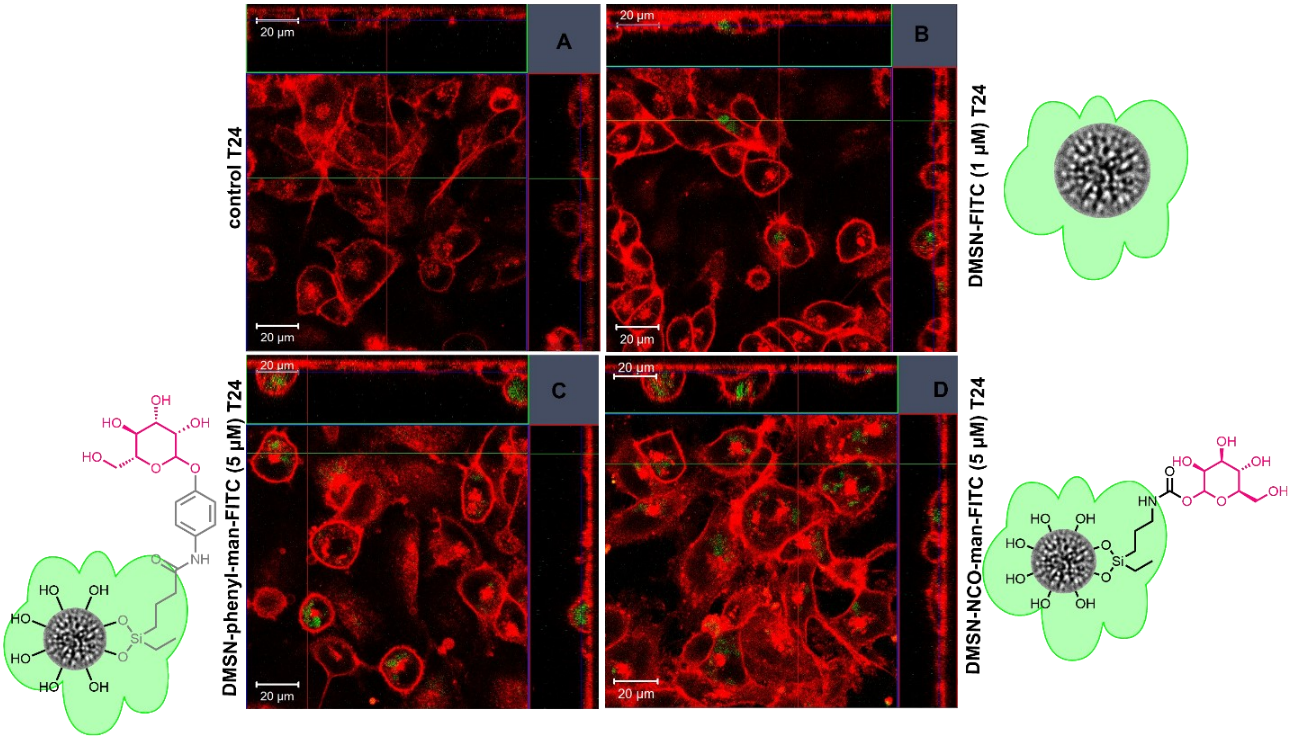

Figure S7. 3D reconstructions and cross sections of live cell fluorescence imaging of T24 cells showing the controls (A), incubation with DMSN-FITC (B; concentration equivalent to 1 µM mannose; 25.7 µg mL^{-1} nanoparticles), DMSN-phenyl-man-FITC (C; mannose concentration of 5 µM), and DMSN-NCO-man-FITC (D; mannose concentration of 5 µM). Scale bars represent 20 µm. The green cloud schematically represents the particle being labeled with FITC.

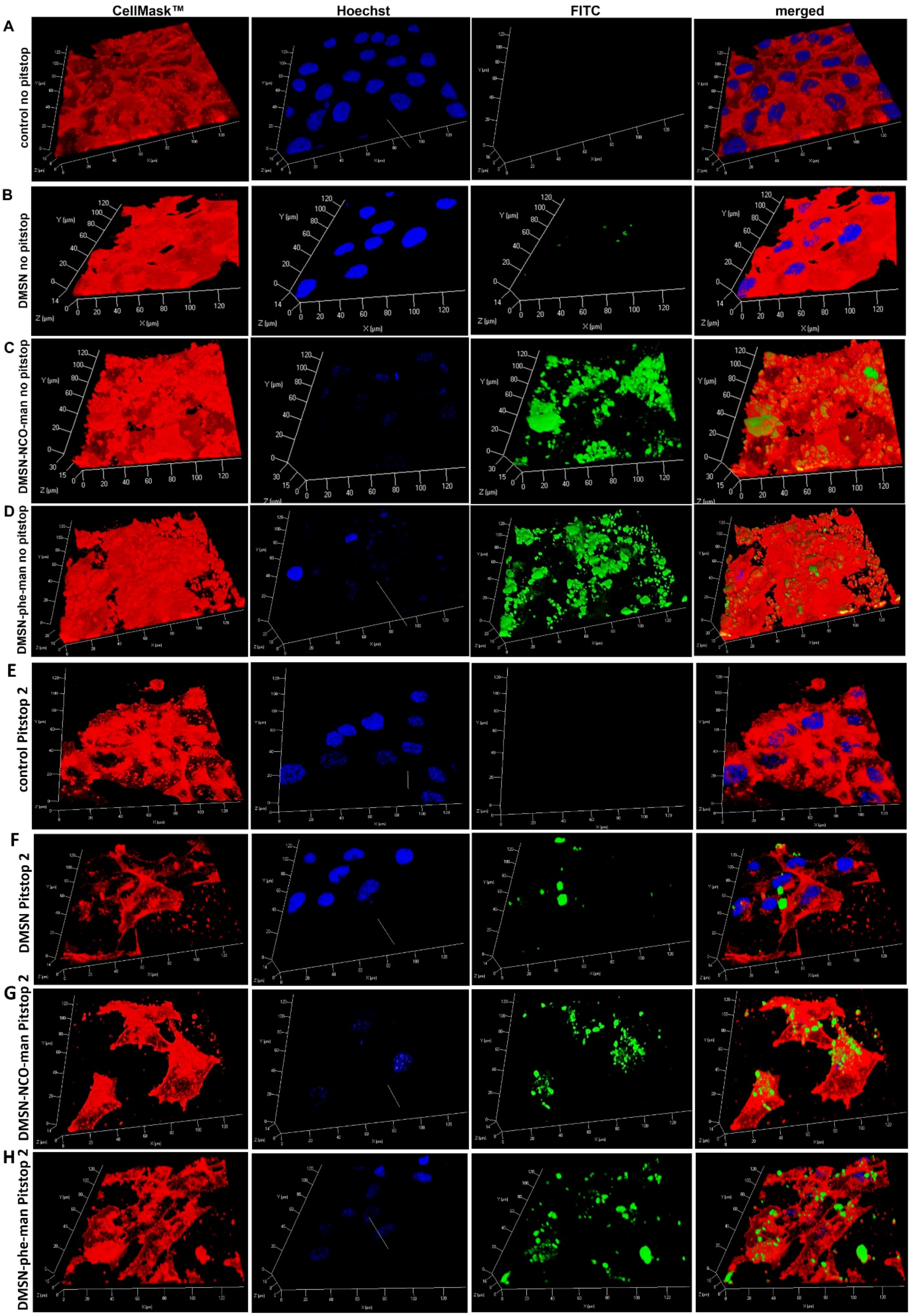
CellMask™
Hoechst
FITC
merged
A
control no pitstop
B
DMSN no pitstop
C
DMSN-NCO-man no pitstop
D
DMSN-phe-man no pitstop
E
control Pitstop 2
F
DMSN Pitstop 2
G
DMSN-NCO-man Pitstop 2
H
DMSN-phe-man Pitstop 2

Figure S8. Representative live cell fluorescence images for the cell uptake experiments of non-functionalized and MANNosylated FITC-labeled silica nanoparticles with T24 cells after 6 h-incubation. Previous treatment without (A, B, C, and D), or with (E, F, G, and H) Pitstop 2. In the 3D reconstructions (63x magnification), the scale bar segmentation is 20 μm and the plasma membrane is represented in red, the fluorescence coming from FITC in green, and the nuclei in blue (mannose concentration of 10 μM, and for non-functionalized DMSNs concentration equivalent to 1 μM mannose, namely 25.7 μg mL^{-1} nanoparticles).

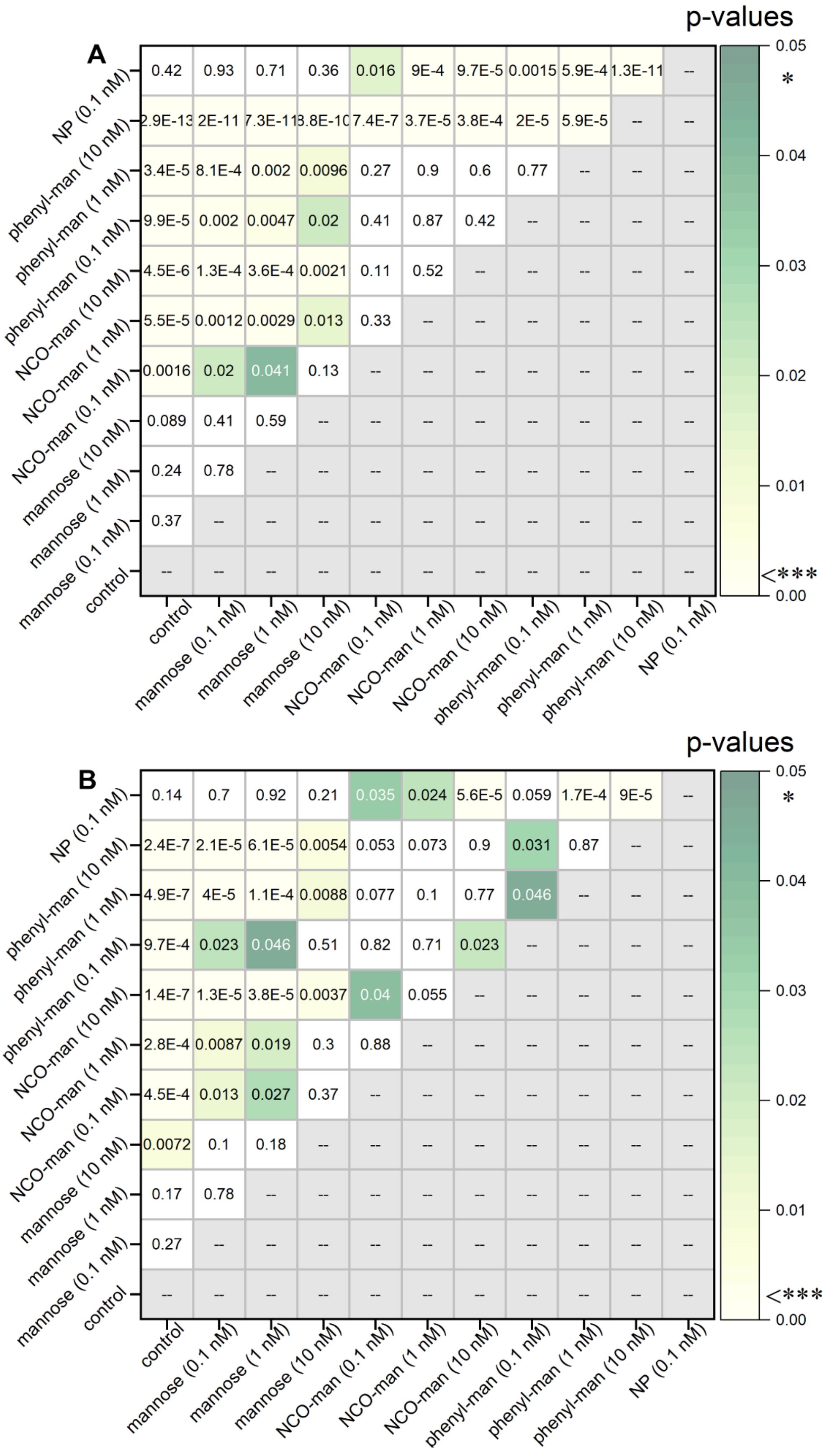
A
p-values
0.05
*
0.04
0.03
0.02
0.01
<***
0.00
NP (0.1 nM)
phenyl-man (10 nM)
phenyl-man (1 nM)
phenyl-man (0.1 nM)
NCO-man (10 nM)
NCO-man (1 nM)
NCO-man (0.1 nM)
mannose (10 nM)
mannose (1 nM)
mannose (0.1 nM)
control
0.42 0.93 0.71 0.36 0.016 9E-4 9.7E-5 0.0015 5.9E-4 1.3E-11
2.9E-13 2E-11 7.3E-11 3.8E-10 7.4E-7 3.7E-5 3.8E-4 2E-5 5.9E-5
3.4E-5 8.1E-4 0.002 0.0096 0.27 0.9 0.6 0.77
9.9E-5 0.002 0.0047 0.02 0.41 0.87 0.42
4.5E-6 1.3E-4 3.6E-4 0.0021 0.11 0.52
5.5E-5 0.0012 0.0029 0.013 0.33
0.0016 0.02 0.041 0.13
0.089 0.41 0.59
0.24 0.78
0.37
control
mannose (0.1 nM)
mannose (1 nM)
mannose (10 nM)
NCO-man (0.1 nM)
NCO-man (1 nM)
NCO-man (10 nM)
phenyl-man (0.1 nM)
phenyl-man (1 nM)
phenyl-man (10 nM)
NP (0.1 nM)
B
p-values
0.05
*
0.04
0.03
0.02
0.01
<***
0.00
NP (0.1 nM)
phenyl-man (10 nM)
phenyl-man (1 nM)
phenyl-man (0.1 nM)
NCO-man (10 nM)
NCO-man (1 nM)
NCO-man (0.1 nM)
mannose (10 nM)
mannose (1 nM)
mannose (0.1 nM)
control
0.14 0.7 0.92 0.21 0.035 0.024 5.6E-5 0.059 1.7E-4 9E-5
2.4E-7 2.1E-5 6.1E-5 0.0054 0.053 0.073 0.9 0.031 0.87
4.9E-7 4E-5 1.1E-4 0.0088 0.077 0.1 0.77 0.046
9.7E-4 0.023 0.046 0.51 0.82 0.71 0.023
1.4E-7 1.3E-5 3.8E-5 0.0037 0.04 0.055
2.8E-4 0.0087 0.019 0.3 0.88
4.5E-4 0.013 0.027 0.37
0.0072 0.1 0.18
0.17 0.78
0.27
control
mannose (0.1 nM)
mannose (1 nM)
mannose (10 nM)
NCO-man (0.1 nM)
NCO-man (1 nM)
NCO-man (10 nM)
phenyl-man (0.1 nM)
phenyl-man (1 nM)
phenyl-man (10 nM)
NP (0.1 nM)

Figure S9. Statistical evaluation of the signal intensity quantification after 24 h of incubation with free mannose, DMSN-NCO-man, and DMSN-phenyl-man (in the concentrations equivalent to 0.1 to 10 μM mannose), and DMSNs (equivalent to 0.1 μM mannose, namely 2.57 μg mL^{-1}). Data are obtained by the quantification of n > 25 regions of interest (ROIs) obtained from three independent cell preparations and expressed as mean fluorescence of relative fluorescence units (r.f.u.). Statistical evaluation of the signal intensity quantification of the TLR4 (A) and CAV-1 (B) was determined via one-way ANOVA with Fisher LSD (* $p < 0.05$; ** $p < 0.01$; *** $p < 0.001$).

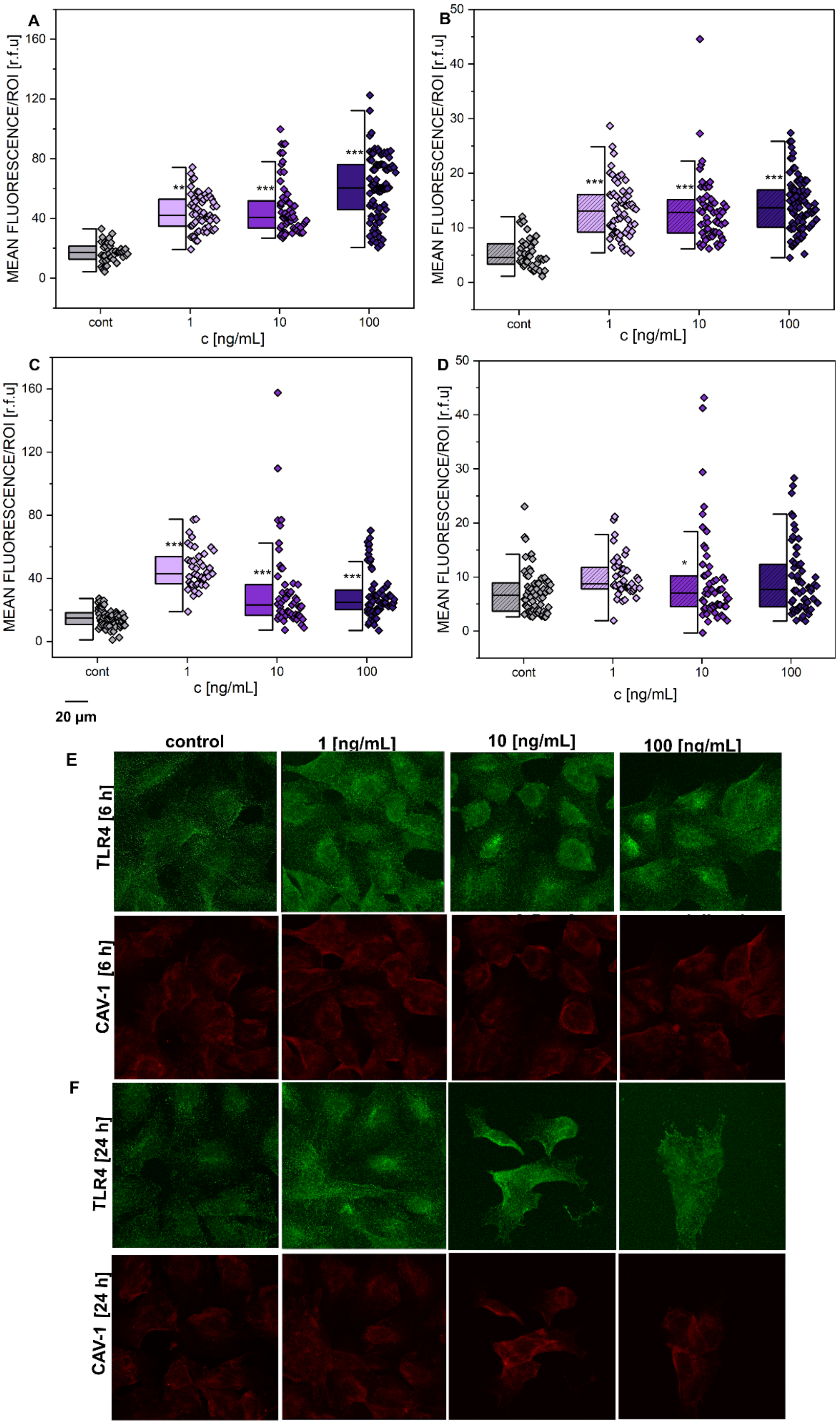
A
B
C
D
E
F
MEAN FLUORESCENCE/ROI [r.f.u]
c [ng/mL]
cont
1
10
100
0
40
80
120
160
10
20
30
40
50
***
**
*
20 µm
control
1 [ng/mL]
10 [ng/mL]
100 [ng/mL]
TLR4 [6 h]
CAV-1 [6 h]
TLR4 [24 h]
CAV-1 [24 h]

Figure S10. Quantification of the signal intensity of the TLR4 (A, C) and CAV-1 (B, D) performed after 6 h (A, B, E) and 24 h (C, D, F) of incubation with LPS, in the concentration range of 1 to 100 ng mL^{-1}. Data are obtained by the quantification of n > 25 region of interest (ROIs) obtained from one cell preparation and expressed as mean fluorescence of relative fluorescence units (r.f.u.). * indicates significant difference in comparison to the control. The complete statistical evaluation can be observed in Figure S11. The significance was determined via one-way ANOVA with Fisher LSD (* $p < 0.05$; ** $p < 0.01$; *** $p < 0.001$).

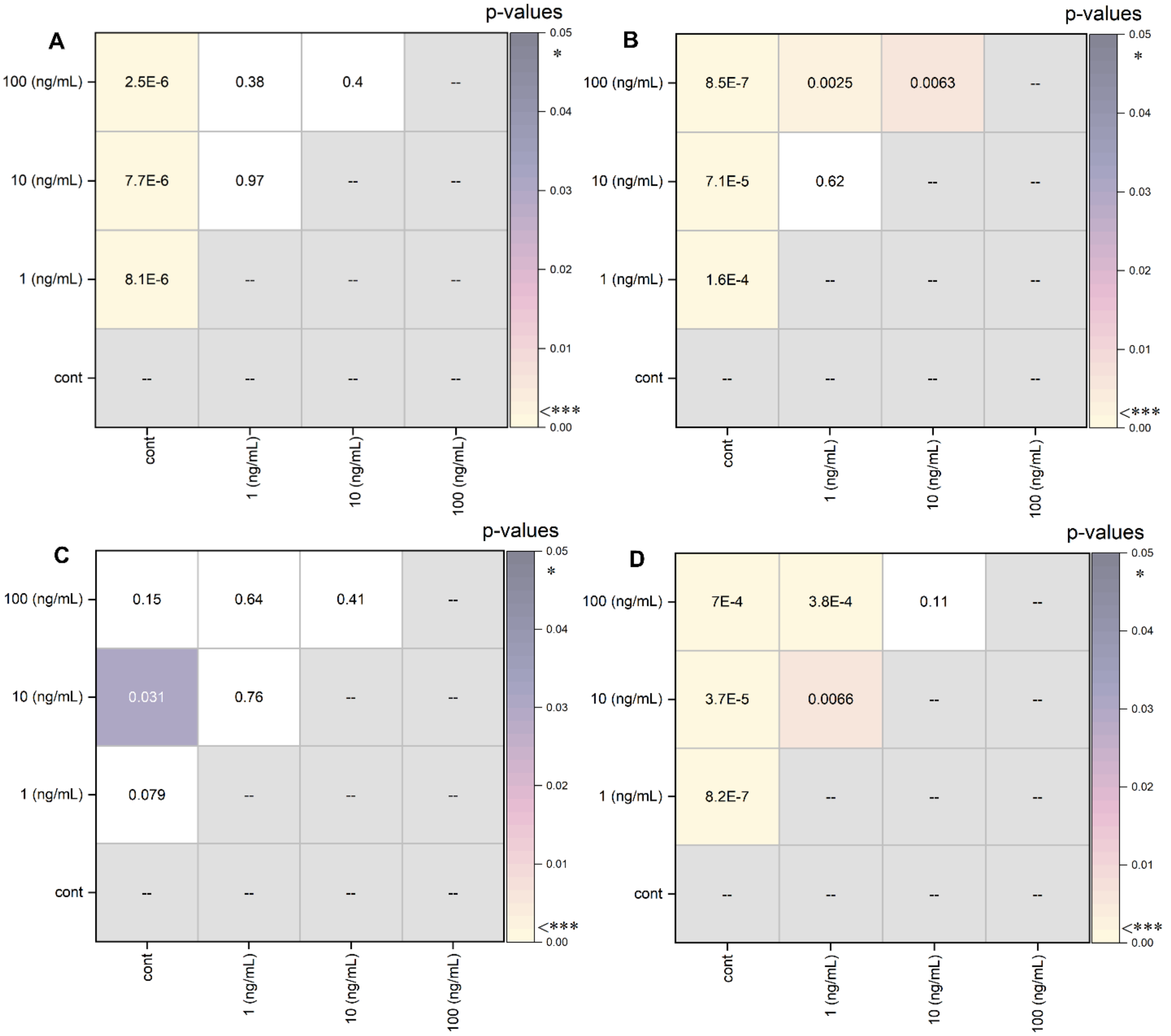

Figure S11. Statistical evaluation of the signal intensity quantification of the CAV-1 (A, C) and TLR4 (B, D). The T24 cell line was incubated with LPS, in the concentration range of 1 to 100 ng mL^{-1} for 6 (A, B) and for 24 h (C, D). Data are obtained by the quantification of n > 25 regions of interest (ROIs) obtained from one cell preparation and expressed as mean fluorescence of relative fluorescence units (r.f.u.). The significance was determined via one-way ANOVA with Fisher LSD (* $p < 0.05$; ** $p < 0.01$; *** $p < 0.001$).

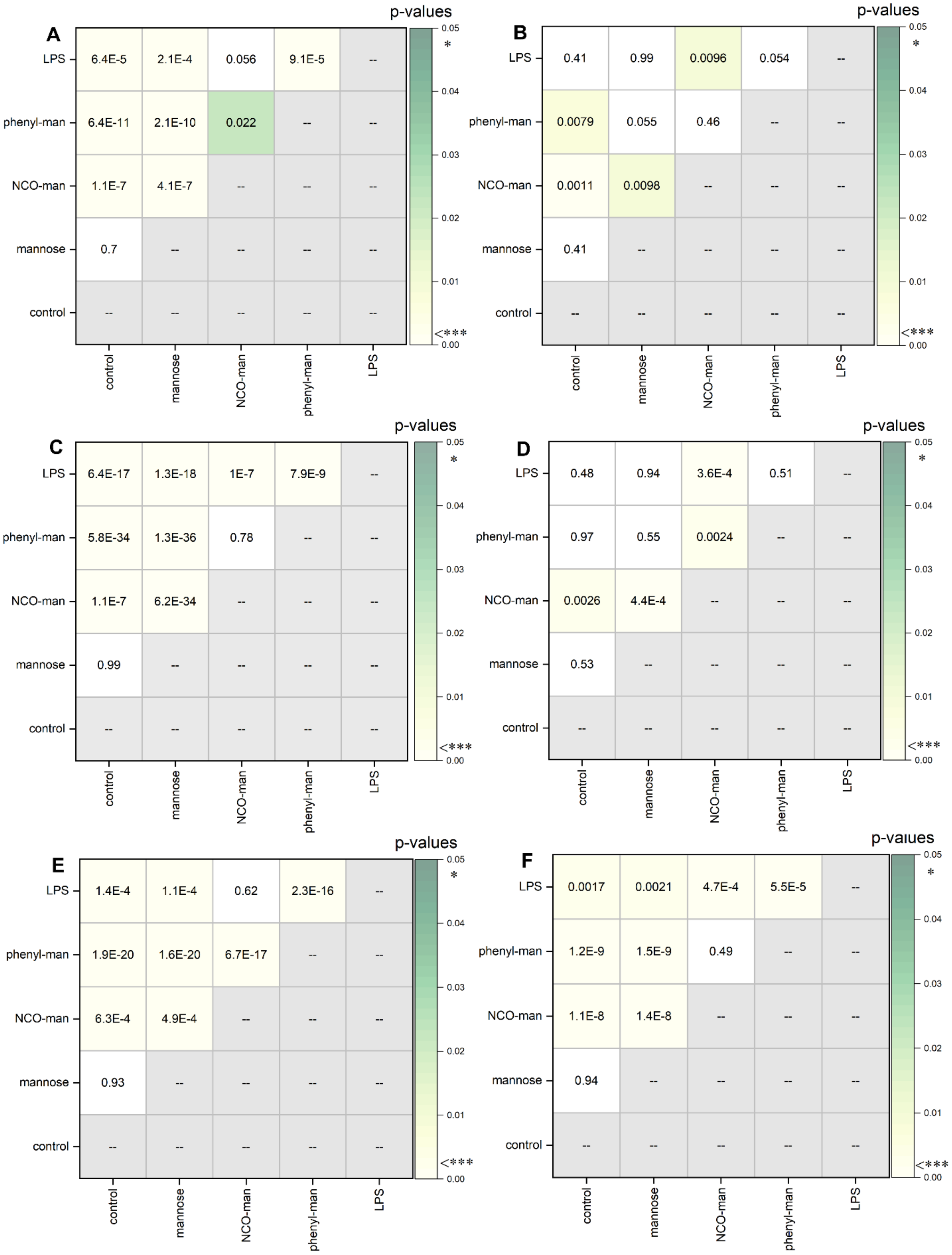
A
p-values
LPS 6.4E-5 2.1E-4 0.056 9.1E-5 --
phenyl-man 6.4E-11 2.1E-10 0.022 -- --
NCO-man 1.1E-7 4.1E-7 -- -- --
mannose 0.7 -- -- -- --
control -- -- -- -- --
control mannose NCO-man phenyl-man LPS
B
p-values
LPS 0.41 0.99 0.0096 0.054 --
phenyl-man 0.0079 0.055 0.46 -- --
NCO-man 0.0011 0.0098 -- -- --
mannose 0.41 -- -- -- --
control -- -- -- -- --
control mannose NCO-man phenyl-man LPS
C
p-values
LPS 6.4E-17 1.3E-18 1E-7 7.9E-9 --
phenyl-man 5.8E-34 1.3E-36 0.78 -- --
NCO-man 1.1E-7 6.2E-34 -- -- --
mannose 0.99 -- -- -- --
control -- -- -- -- --
control mannose NCO-man phenyl-man LPS
D
p-values
LPS 0.48 0.94 3.6E-4 0.51 --
phenyl-man 0.97 0.55 0.0024 -- --
NCO-man 0.0026 4.4E-4 -- -- --
mannose 0.53 -- -- -- --
control -- -- -- -- --
control mannose NCO-man phenyl-man LPS
E
p-values
LPS 1.4E-4 1.1E-4 0.62 2.3E-16 --
phenyl-man 1.9E-20 1.6E-20 6.7E-17 -- --
NCO-man 6.3E-4 4.9E-4 -- -- --
mannose 0.93 -- -- -- --
control -- -- -- -- --
control mannose NCO-man phenyl-man LPS
F
p-values
LPS 0.0017 0.0021 4.7E-4 5.5E-5 --
phenyl-man 1.2E-9 1.5E-9 0.49 -- --
NCO-man 1.1E-8 1.4E-8 -- -- --
mannose 0.94 -- -- -- --
control -- -- -- -- --
control mannose NCO-man phenyl-man LPS
0.05 * 0.04 0.03 0.02 0.01 <*** 0.00

Figure S12. Statistical evaluation of the signal intensity quantification of the TLR4 (A, C, E) and CAV-1 (B, D, F) for the experiment mimicking an infection via co-incubation (material + LPS) (A and B), the presence of the material followed by the pro-inflammatory treatment: 1st material, 2nd LPS (C and D), and finally, 1st LPS and 2nd material (E and F). Incubations (24 h) were carried out with free mannose, DMSN-NCO-man, and DMSN-phenyl-man (in the concentration of 10 μM mannose), and LPS (1 ng mL^{-1}). Data are obtained by the quantification of $n > 25$ regions of interest (ROIs) obtained from three independent cell preparations and expressed as mean fluorescence of relative fluorescence units (r.f.u.). The significance was determined via one-way ANOVA with Fisher LSD (* $p < 0.05$; ** $p < 0.01$; *** $p < 0.001$).

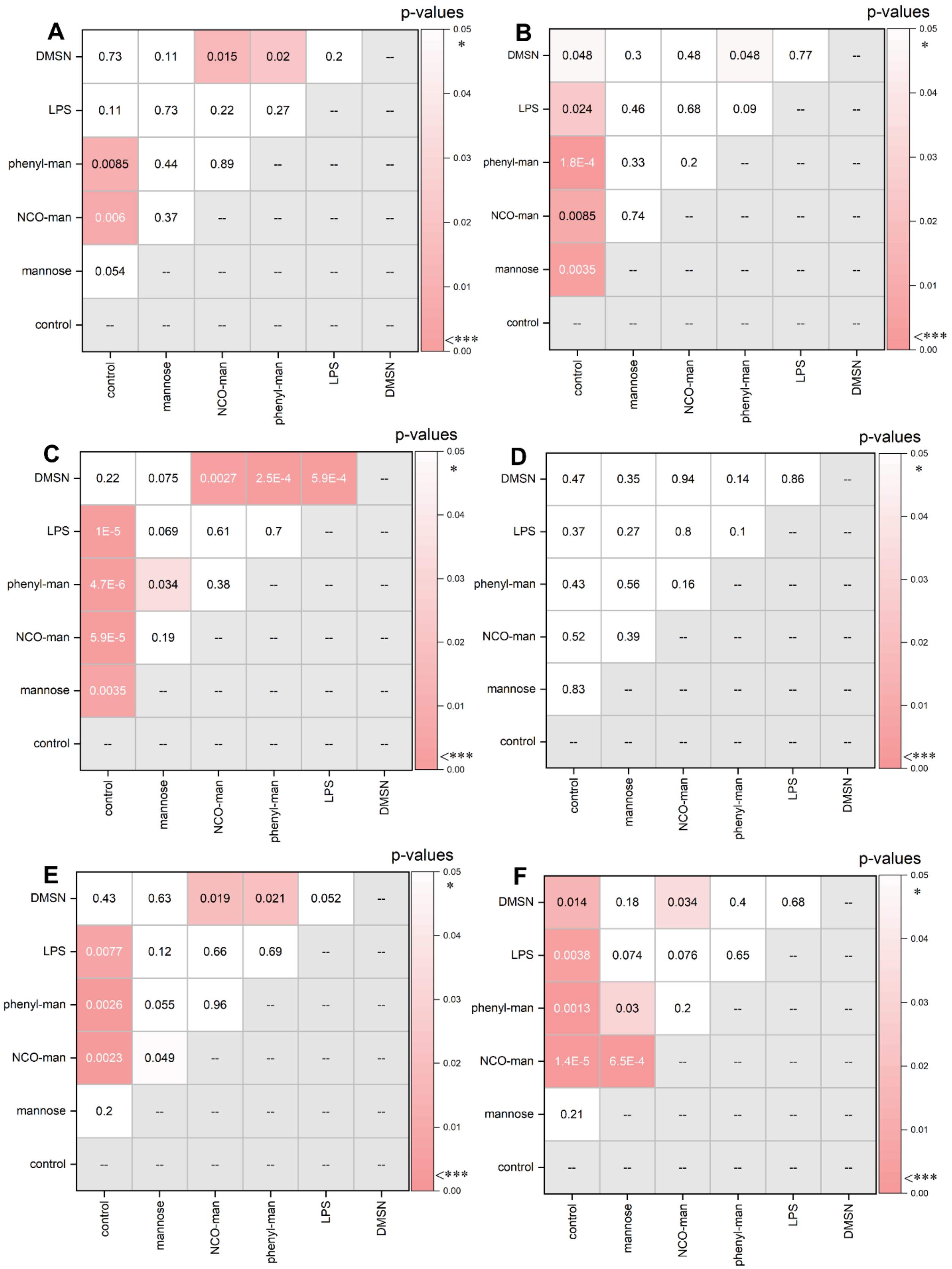
A
p-values
DMSN 0.73 0.11 0.015 0.02 0.2 --
LPS 0.11 0.73 0.22 0.27 -- --
phenyl-man 0.0085 0.44 0.89 -- -- --
NCO-man 0.006 0.37 -- -- -- --
mannose 0.054 -- -- -- -- --
control -- -- -- -- -- --
control mannose NCO-man phenyl-man LPS DMSN
0.05 * 0.04 0.03 0.02 0.01 <*** 0.00
B
p-values
DMSN 0.048 0.3 0.48 0.048 0.77 --
LPS 0.024 0.46 0.68 0.09 -- --
phenyl-man 1.8E-4 0.33 0.2 -- -- --
NCO-man 0.0085 0.74 -- -- -- --
mannose 0.0035 -- -- -- -- --
control -- -- -- -- -- --
control mannose NCO-man phenyl-man LPS DMSN
0.05 * 0.04 0.03 0.02 0.01 <*** 0.00
C
p-values
DMSN 0.22 0.075 0.0027 2.5E-4 5.9E-4 --
LPS 1E-5 0.069 0.61 0.7 -- --
phenyl-man 4.7E-6 0.034 0.38 -- -- --
NCO-man 5.9E-5 0.19 -- -- -- --
mannose 0.0035 -- -- -- -- --
control -- -- -- -- -- --
control mannose NCO-man phenyl-man LPS DMSN
0.05 * 0.04 0.03 0.02 0.01 <*** 0.00
D
p-values
DMSN 0.47 0.35 0.94 0.14 0.86 --
LPS 0.37 0.27 0.8 0.1 -- --
phenyl-man 0.43 0.56 0.16 -- -- --
NCO-man 0.52 0.39 -- -- -- --
mannose 0.83 -- -- -- -- --
control -- -- -- -- -- --
control mannose NCO-man phenyl-man LPS DMSN
0.05 * 0.04 0.03 0.02 0.01 <*** 0.00
E
p-values
DMSN 0.43 0.63 0.019 0.021 0.052 --
LPS 0.0077 0.12 0.66 0.69 -- --
phenyl-man 0.0026 0.055 0.96 -- -- --
NCO-man 0.0023 0.049 -- -- -- --
mannose 0.2 -- -- -- -- --
control -- -- -- -- -- --
control mannose NCO-man phenyl-man LPS DMSN
0.05 * 0.04 0.03 0.02 0.01 <*** 0.00
F
p-values
DMSN 0.014 0.18 0.034 0.4 0.68 --
LPS 0.0038 0.074 0.076 0.65 -- --
phenyl-man 0.0013 0.03 0.2 -- -- --
NCO-man 1.4E-5 6.5E-4 -- -- -- --
mannose 0.21 -- -- -- -- --
control -- -- -- -- -- --
control mannose NCO-man phenyl-man LPS DMSN
0.05 * 0.04 0.03 0.02 0.01 <*** 0.00

Figure S13. Statistical evaluation of the signal intensity quantification of the NF-κB protein in the cytoplasm (A, C, E) and nucleus (B, D, F) after 3 h (A, B), 6 h (C, D) and 24 h (E, F). Incubations were carried out with free mannose, DMSN-NCO-man, and DMSN-phenyl-man (in the concentration of 10 μM mannose), LPS (1 ng mL^{-1}), and non-functionalized DMSNs which were applied at a concentration equivalent to 0.1 μM mannose (2.57 μg mL^{-1}). Data are obtained by the quantification of $n > 25$ regions of interest (ROIs) obtained from three independent cell preparations and expressed as mean fluorescence of relative fluorescence units (r.f.u.). The significance was determined via one-way ANOVA with Fisher LSD (* $p < 0.05$; ** $p < 0.01$; *** $p < 0.001$).
